# Chromium-catalyzed cyclopropanation of alkenes with bromoform in the presence of 2,3,5,6-tetramethyl-1,4-bis(trimethylsilyl)-1,4-dihydropyrazine†

**DOI:** 10.1039/d0sc00964d

**Published:** 2020-03-11

**Authors:** Hideaki Ikeda, Kohei Nishi, Hayato Tsurugi, Kazushi Mashima

**Affiliations:** Department of Chemistry, Graduate School of Engineering Science, Osaka University Toyonaka Osaka 560-8531 Japan mashima@chem.es.osaka-u.ac.jp tsurugi@chem.es.osaka-u.ac.jp

## Abstract

Chromium-catalyzed cyclopropanation of alkenes with bromoform was achieved to produce the corresponding bromocyclopropanes. In this catalytic cyclopropanation, an organosilicon reductant, 2,3,5,6-tetramethyl-1,4-bis(trimethylsilyl)-1,4-dihydropyrazine (**1a**), was indispensable for reducing CrCl_3_(thf)_3_ to CrCl_2_(thf)_3_, as well as for *in situ* generation of (bromomethylidene)chromium(iii) species from (dibromomethyl)chromium(iii) species. The (bromomethylidene)chromium(iii) species are proposed to react spontaneously with alkenes to give the corresponding bromocyclopropanes. This catalytic cyclopropanation was applied to various olefinic substrates, such as allyl ethers, allyl esters, terminal alkenes, and cyclic alkenes.

## Abstract 2

Chromium-catalyzed cyclopropanation of alkenes with bromoform was achieved to produce the corresponding bromocyclopropanes.

## Graphical abstract

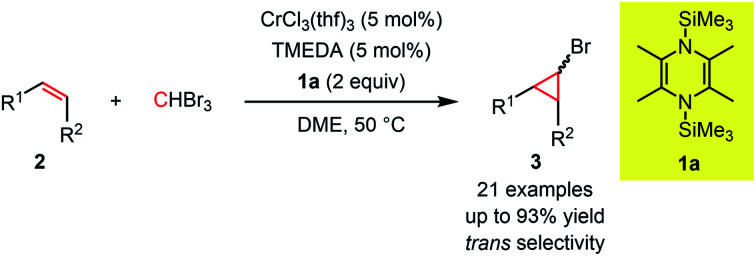

## Introduction

**Fig. 1:**
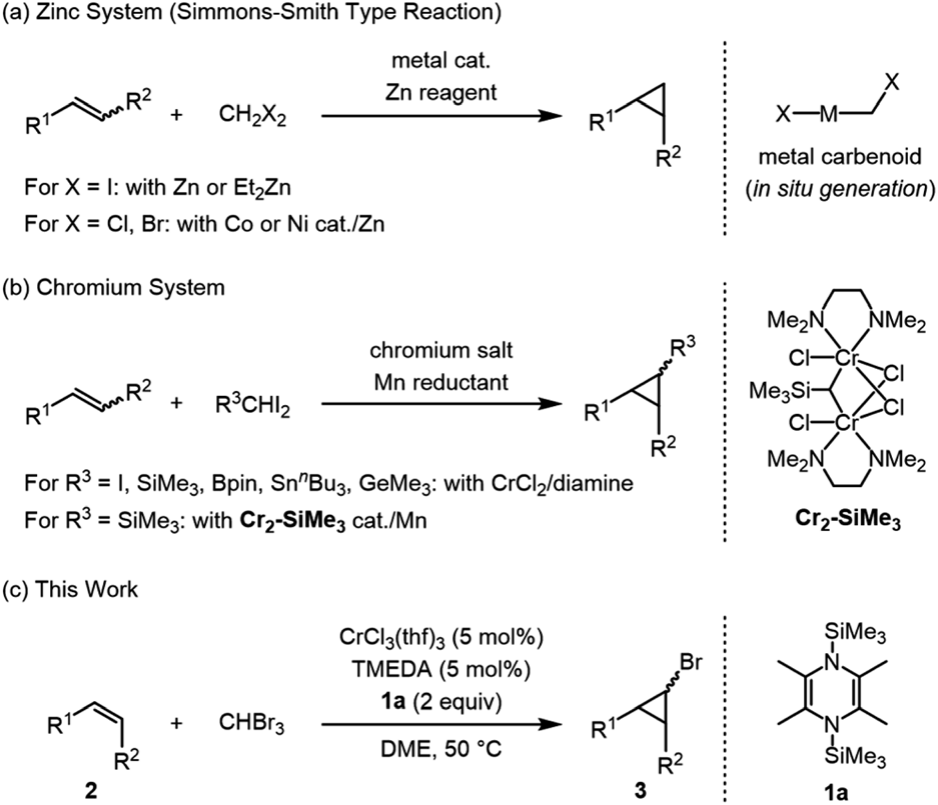
Metal-assisted cyclopropanation of alkenes with di- and trihalomethanes; (a) cyclopropanation with excess zinc powder, (b) cyclopropanation with excess or catalytic amounts of chromium, and (c) bromocyclopropanation with catalytic amounts of chromium and organosilicon reductant **1a** (This Work).

Cyclopropane is a strained three-membered carbocycle, and a common structural motif in pharmaceutical and biologically active compounds.^1^ The synthesis of cyclopropanes from easily available starting materials is in high demand, and several stoichiometric synthetic protocols for the C3 ring have been developed: (1) classical reductive cyclization of 1,3-dihalopropanes or β-haloalkenes using metal-based reductants such as lithium and magnesium,^2^ (2) cyclopropanation of alkenes using haloform (CHX_3_) and a strong base in phase-transfer conditions to afford geminal dihalocyclopropanes,^3^ and (3) cyclopropanation of alkenes using nitrogen-, phosphonium-, and sulfur-ylides,^4^*in situ*-generated zinc carbenoid from Zn reagents and CH_2_I_2_ (Simmons–Smith reaction),^5^ and *in situ*-generated chromium carbene species from excess amounts of CrCl_2_, diamine ligands, and RCHI_2_.^6^ In contrast to these stoichiometric reactions, metal-catalyzed cyclopropanation of alkenes using diazomethane and its derivatives is an alternative effective protocol, despite the use of explosive diazomethane derivatives.^7^ To avoid the use of explosive compounds, the development of metal-catalyzed cyclopropanation reactions using non-explosive reagents was recently explored.^8^ Uyeda *et al.* reported that some nickel and cobalt complexes serve as catalysts for Simmons–Smith type reactions of alkenes with less reactive CH_2_Cl_2_ and CH_2_Br_2_ in the presence of excess zinc powder (Fig. 1a).^8*f*–8*i*^ Takai *et al.* reported that chromium-catalyzed cyclopropanation of alkenes with Me_3_SiCHI_2_ proceeds in the presence of catalytic amounts of chromium complex and excess Mn powder as a reducing reagent, from which *gem*-dichromiomethane complexes (**Cr2–SiMe3**) are isolated (Fig. 1b),^9*a*^ and, similarly, Anwander *et al.* isolated an iodomethyl-bridged dichromium complex by treating CrCl_2_ with CHI_3_ as a key intermediate species for cyclopropanation to give iodocyclopropanes.^9*b*^

We have focused our attention on the versatility of a family of organosilicon-based reductants, 1,4-bis(trimethylsilyl)-1,4-dihydropyrazine derivatives and 1,1′-bis(trimethylsilyl)-4,4′-bipyridinylidene, as stoichiometric reagents for reducing not only transition metal complexes^10^ for reductive C–C bond formation without generating any metal-based waste,^10*b*,10*d*^ but also main group halides for producing multiple bonds between main group elements^11^ and some oxo compounds, such as nitrobenzenes and sulfoxides, in a metal-free fashion to give respectively anilines and thioethers.^12*c*,12*d*^ Herein, we report that chromium(iii) trichloride with *N*,*N*,*N*′,*N*′-tetramethylethylenediamine (TMEDA) served as a catalyst for the cyclopropanation of alkenes with bromoform in combination with 2,3,5,6-tetramethyl-1,4-bis(trimethylsilyl)-1,4-dihydropyrazine (**1a**) to obtain synthetically useful bromocyclopropanes in high yield (Fig. 1c).^13^

## Results and discussion

We then screened conditions by tuning reductants, additives, and supporting ligands to optimize the chromium-catalyzed cyclopropanation of allyl benzyl ether (**2a**) with bromoform as a model reaction, and the results are summarized in Table 1. When we used a 1 : 1 mixture of CrCl_3_(thf)_3_ (5 mol%) and TMEDA (5 mol%) in the presence of **1a** (2 equiv.) in 1,2-dimethoxyethane (DME) at 50 °C for 24 h, bromocyclopropane **3a** was obtained in 98% yield with high *trans* (89%) selectivity (entry 1). Cyclopropanation at 25 °C resulted in a slightly lower yield (81%) of **3a** with almost the same *trans* selectivity (entry 1 *vs.* 2). No cyclopropanation product was obtained when organosilicon compounds **1b–d** were used as the reducing reagents (entries 3–5), although **1b–d** reduced CrCl_3_(thf)_3_ to CrCl_2_, probably due to coordination of the reduction byproducts, 2,5-dimethylpyrazine (from **1b**), pyrazine (from **1c**), and 4,4′-bipyridyl (from **1d**), to the chromium center, as confirmed by the inhibition of the catalytic reaction when pyrazine was added under the standard conditions. Screening of several multidentate nitrogen-based ligands revealed that TMEDA was the best ligand for this catalytic reaction (entry 1 *vs.* 12–17; amines, phosphines, and other ligands in ESI†). Notably, no reaction was observed when using typical organic and inorganic reductants, such as tetrakis(dimethylamino)ethylene (TDAE), Zn, and Mn powder (entries 6–8). The coordination of TMEDA to the chromium center was essentially required to produce the catalytic activity: the addition of either ZnCl_2_ (2 equiv.) or MnCl_2_ (2 equiv.) to the standard reaction conditions resulted in no reaction (entry 9) or lowered the yield of **3a** (entry 10), respectively, due to the removal of TMEDA from the chromium center,^9*a*^ while under ligand-free conditions, the yield of **3a** decreased significantly (entry 11). When isolated CrCl_3_(tmeda) (5 mol%) was used as the catalyst, the yield of **3a** was comparable with that of the *in situ* CrCl_3_(thf)_3_ and TMEDA system (entry 18).

**Table 1:** Optimization study of reaction conditions^a^

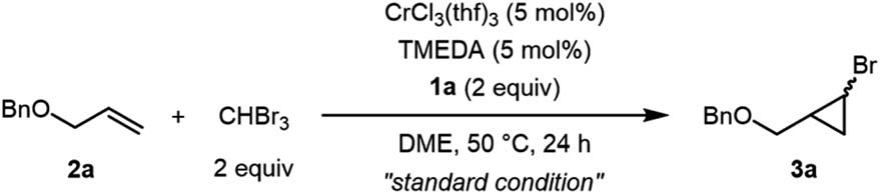			
Entry	Variation from standard condition	Yield (%)^b^	*trans* : *cis*^b^
1	None	98 (93)^c^	89 : 11
2	25 °C, 24 h	81	90 : 10
3	**1b** (2 equiv.) instead of **1a**	0	N/A
4	**1c** (2 equiv.) instead of **1a**	0	N/A
5	**1d** (2 equiv.) instead of **1a**	0	N/A
6	TDAE (2 equiv.) instead of **1a**	0	N/A
7	Zn (6 equiv.) instead of **1a**	0	N/A
8	Mn (6 equiv.) instead of **1a**	0	N/A
9	Addition of ZnCl_2_ (2 equiv.)	0	N/A
10	Addition of MnCl_2_ (2 equiv.)	56	87 : 13
11	Without TMEDA	7	71 : 29
12	**L1** (5 mol%) instead of TMEDA	97	90 : 10
13	**L2** (5 mol%) instead of TMEDA	7	57 : 43
14	**L3** (5 mol%) instead of TMEDA	0	N/A
15	**L4** (5 mol%) instead of TMEDA	8	>99 : 1
16	**L5** (5 mol%) instead of TMEDA	0	N/A
17	**L6** (5 mol%) instead of TMEDA	0	N/A
18	CrCl_3_(tmeda) (5 mol%) instead of CrCl_3_(thf)_3_ and TMEDA	90	88 : 12
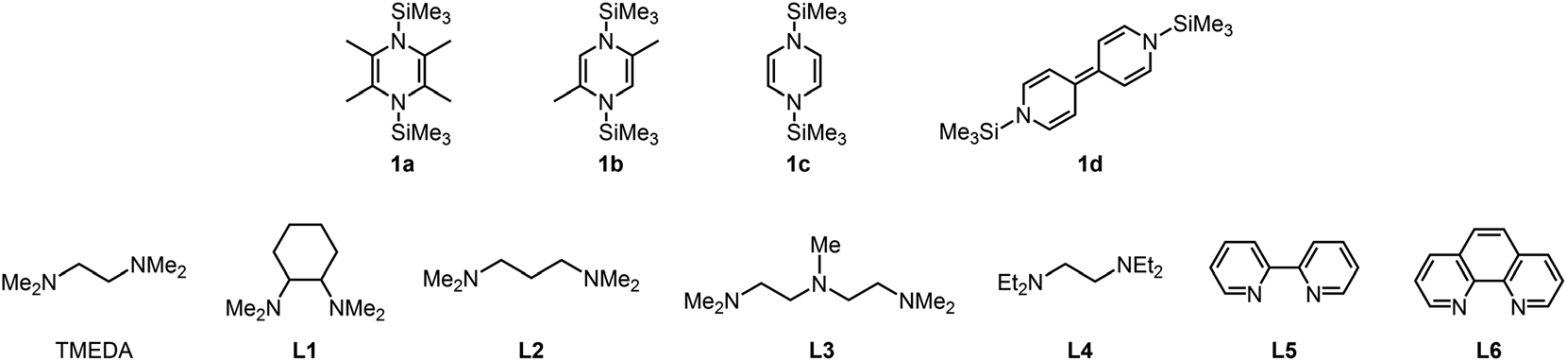			

a Reaction condition: **2a** (0.1 mmol), bromoform (2 equiv.), CrCl_3_(thf)_3_ (5 mol%), ligand (5 mol%), and reductant (above-mentioned amount) in 1,2-dimethoxyethane (DME, 0.10 M) at 50 °C for 24 hours.

b Determined by ^1^H NMR analysis using 1,3,5-trimethoxybenzene as an internal standard.

c Isolated yield. TDAE: tetrakis(dimethylamino)ethylene.

With the optimized reaction conditions in hand, we analyzed the substrate scope of the alkenes (Table 2). Allyl phenyl ether (**2b**) was converted to the corresponding bromocyclopropane **3b** in 92% yield with high *trans* selectivity. Other allyl aryl ethers **2c–g** with electron-withdrawing and -donating substituents on the phenyl ring were transformed to the corresponding cyclopropanes **3c–g** in moderate to high yields, with a cyano group or halogen atoms at the *para*-position of the aryl ring remaining intact during the cyclopropanation reaction. Reaction of CHBr_3_ with allyl butyl ether (**2h**) afforded **3h** in 81% yield with a *trans* : *cis* ratio of 87 : 13. The carbonyl group also tolerated the reductive conditions to produce cyclopropanes; benzoyl-substituted alkene **2i** was converted to **3i** in 75% yield, while allyl carbonate **2j**, which is typically used for allylic substitution of nucleophiles, afforded **3j** in 60% yield without any decomposition of **2j**. Allylamine **2k** was also applicable and the corresponding cyclopropylmethylamine **3k** was obtained in 64% yield. Simple α-olefins, such as allylbenzene **2l**, 5-hexenyl acetate **2m**, 1-octene **2n**, and vinylcyclohexane **2o**, gave the corresponding cyclopropanes **3l–o** in good yield. When we applied substrates possessing two olefinic moieties, a terminal and mono-substituted olefinic part was selectively cyclopropanated to give **3p** and **3q** in moderate yield. Internal alkenes with *cis*-configuration were also applicable to our catalytic system: *cis*-1,4-diacetoxy-2-butene (**2r**) showed a moderate reactivity to give the corresponding cyclopropane **3r** in 47% yield, while some cyclic alkenes such as cycloheptene (**2s**), cyclooctene (**2t**) and acenaphthylene (**2u**) were applicable to afford polycyclic compounds **3s**, **3t**, and **3u** in moderate to high yields, though debromination of initially formed bromocyclopropane might be involved for the formation of **3u**. Other olefins such as styrene, 1,1-disubstituted alkenes, acyclic internal alkenes, and dienes were not applicable in this cyclopropanation reaction (see ESI† for the limitations of this cyclopropanation).

**Table 2:** Scope of substrates^a^

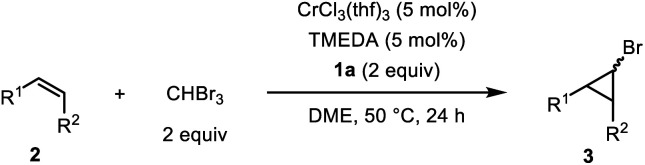
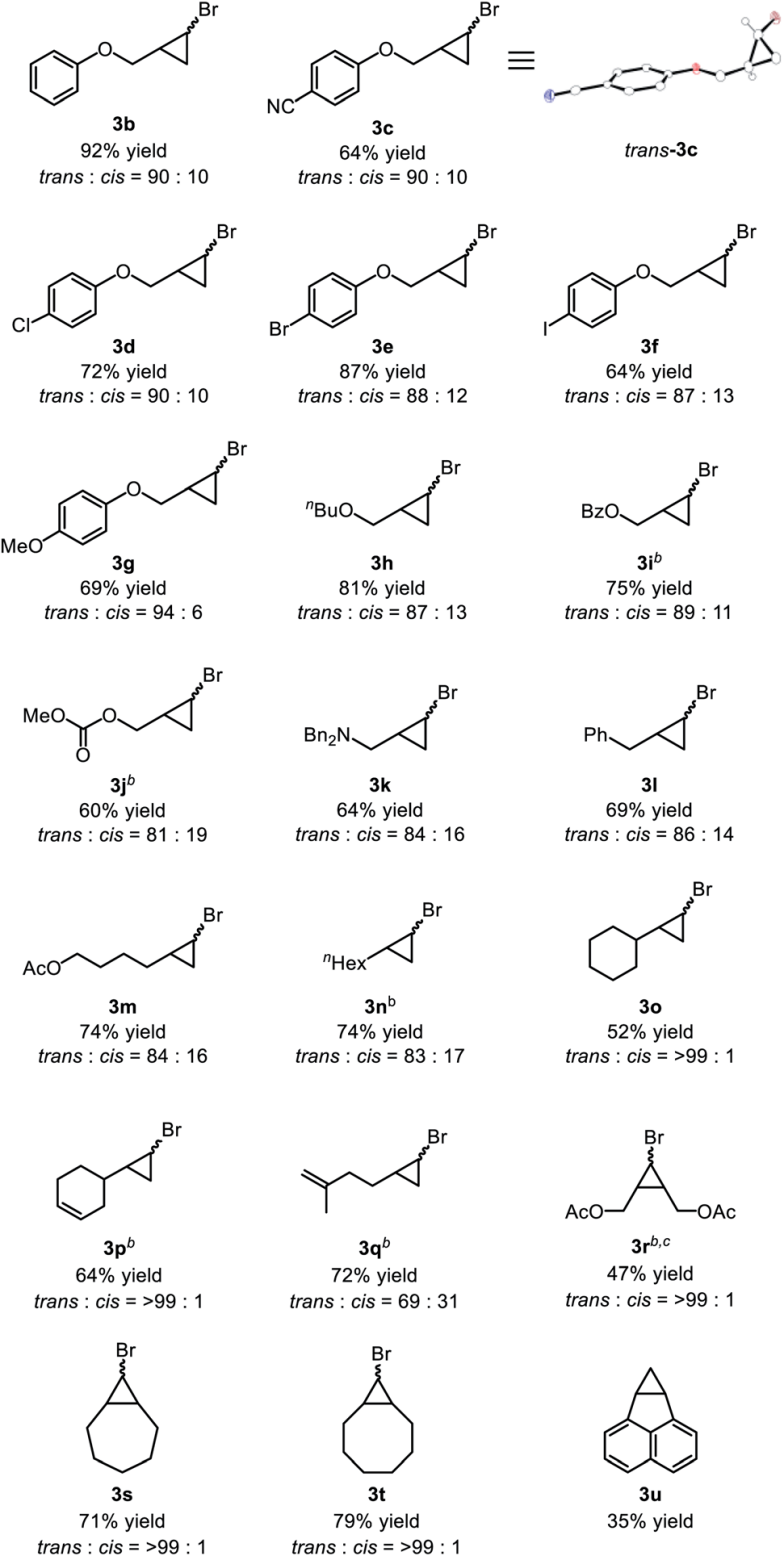

a Standard reaction condition: **2** (0.4 mmol), bromoform (0.8 mmol, 2 equiv.), CrCl_3_(thf)_3_ (0.02 mmol, 5 mol%), TMEDA (5 mol%), and **1a** (0.8 mmol, 2 equiv.) in 1,2-dimethoxyethane (DME, 4 mL) at 50 °C for 24 hours.

b CrCl_3_(thf)_3_/TMEDA: 10 mol%.

c NMR yield. Isolated yields after purification by flash column chromatography are noted.

**Scheme 1:**
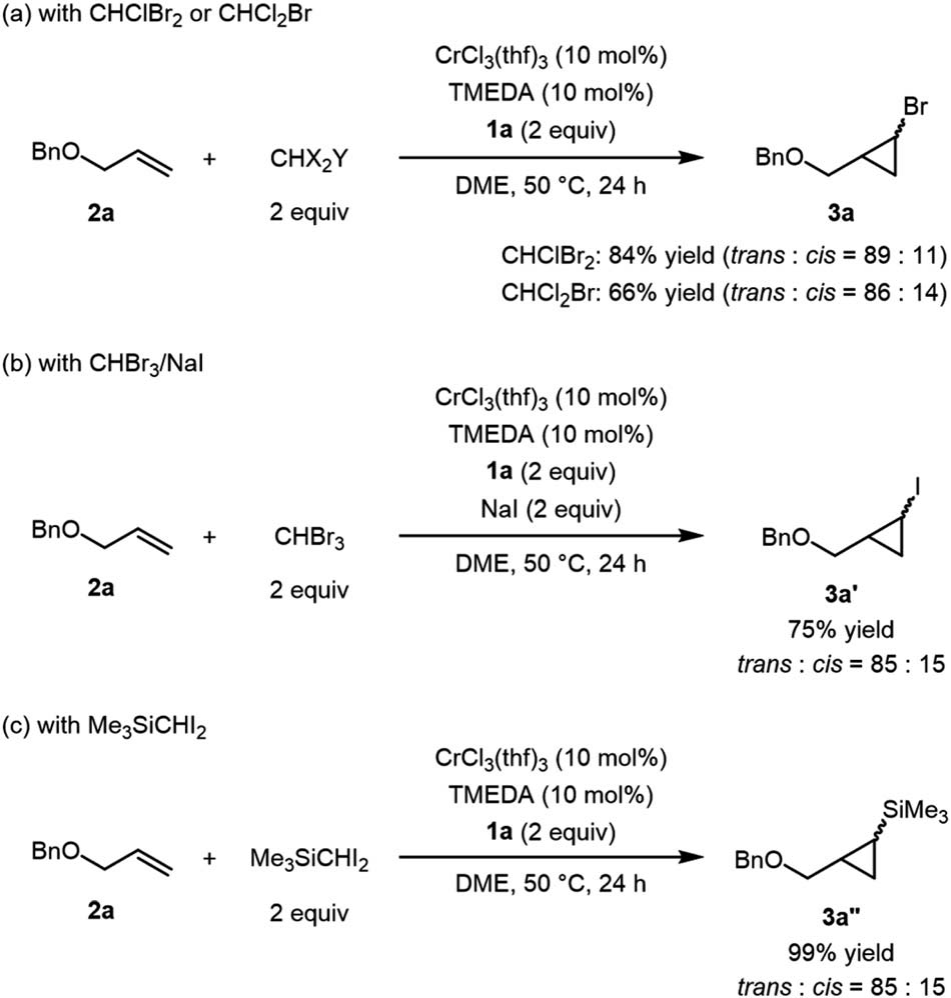
Cyclopropanation using tri- and dihalomethanes. (a) Reaction with CHClBr_2_ and CHCl_2_Br. (b) Reaction with CHBr_3_ in the presence of NaI. (c) Reaction with Me_3_SiCHI_2_.

In addition to bromoform, other trihalomethanes were applicable to the catalytic cyclopropanation. It was noteworthy that, in the reactions of **2a** with both CHClBr_2_ and CHCl_2_Br, the same bromocyclopropane **3a** was obtained as the major product in 84% and 66% yield, respectively, along with chlorocyclopropane as a minor product, although it was much easier to cleave the C–Br bond than the C–Cl bond (Scheme 1a).^14^ Direct synthesis of iodocyclopropane was not accessible under the optimized conditions with CHI_3_, while cyclopropanation using CHBr_3_ in the presence of NaI (2 equiv.) produced iodocyclopropane **3a′** instead of **3a** in 75% yield (Scheme 1b). When Me_3_SiCHI_2_ was used as a C1 source, corresponding silyl-substituted cyclopropane **3a′′** was obtained in quantitative yield (Scheme 1c).

To elucidate the reaction mechanism, we carried out a kinetic study for the formation of **3a**, and the resulting data were analyzed by variable time normalization analysis (see ESI†).^15^ The overall reaction rate did not change with various concentrations of chromium catalyst (0.004–0.01 M) and alkene **2a** (0.08–0.12 M), giving a rate dependence of [Cr]^0^[**2a**]^0^, which is in sharp contrast to the report of Takai *et al.* who found that chromium-catalyzed cyclopropanation with Me_3_SiCHI_2_ obeys first-order dependence on the concentrations of both a chromium carbene complex and **2a**, giving a rate dependence of [Cr]^1^[**2a**]^1^.^9*a*^ Such a difference was further observed in the reaction profile; no induction period was observed under the various reaction conditions.^16^

**Fig. 2:**
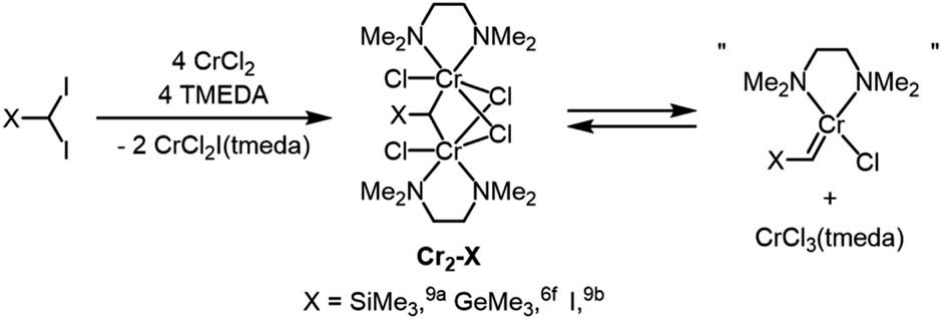
Proposed reaction pathway for chromium carbene species from isolated dichromium complexes by Takai *et al.* and Anwander *et al.*

Next, to understand how **1a** functioned to generate a catalytically active species, we performed some control experiments. Direct activation of CHBr_3_ by **1a** was excluded because no significant rate acceleration was observed when a mixture of CHBr_3_ and **1a** was pre-treated by stirring at 50 °C for 1 hour before adding the chromium catalyst (see ESI†). Although we tried repeatedly to isolate the dichromium species having a bridging bromomethyl group, the target complex could not be isolated and characterized, probably due to the instability of the bromomethyl-bridged dichromium species (see ESI†). In previous reports, however, *gem*-dichromiomethane complexes (**Cr2–X**) was isolated as key intermediates prior to generating reactive mononuclear carbene species *via* disproportionation (Fig. 2). Takai *et al.* reported the first example of an isolated *gem*-dichromiomethane complex (**Cr2–SiMe3**) by introducing a bulky trimethylsilyl-substituent on a carbon atom of diiodomethane, from which silylcyclopropanes were obtained upon treatment with alkenes. The related germanium derivative, **Cr2–GeMe3**, was also isolated and used for cyclopropanation. Anwander *et al.* independently observed the formation of a *gem*-dichromiomethane complex (**Cr2–I**) from the reaction of CrCl_2_ and CHI_3_ at low temperature.

**Scheme 2:**
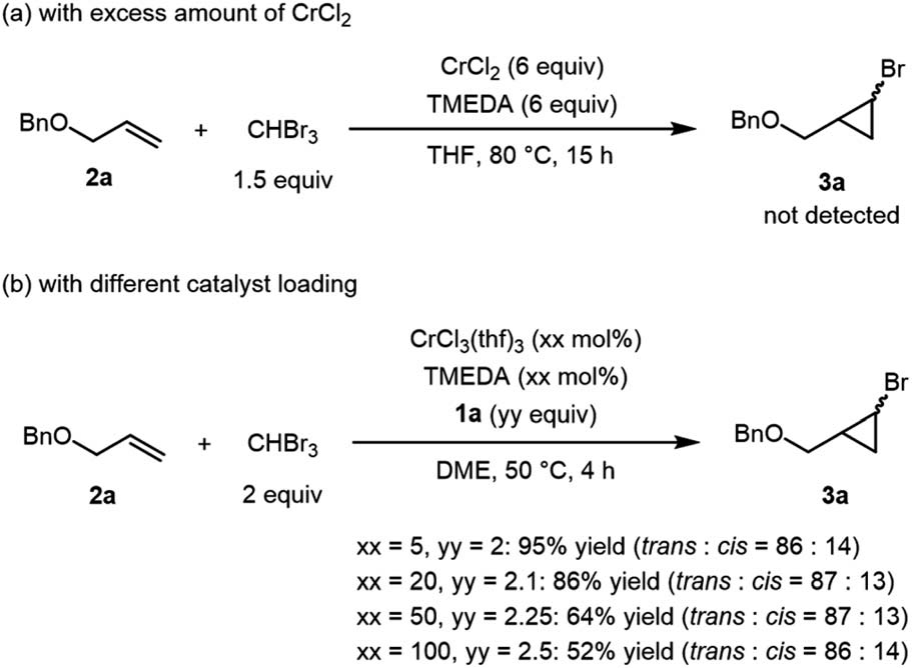
Control experiments. (a) Reaction in the presence of excess amount of CrCl_2_. (b) Reaction with different catalyst loading.

We next conducted a stoichiometric cyclopropanation reaction of alkene **2a** with bromoform in the presence of excess CrCl_2_ (Scheme 2). The desired cyclopropane **3a** was not obtained even at 80 °C (Scheme 2a), although formation of the corresponding cyclopropanes was observed when iodoform and diiodomethane derivatives were used. Moreover, under the catalytic conditions using **1a**, the yield of **3a** gradually decreased as the catalyst loading was increased from 5 to 100 mol% (Scheme 2b). The lower product yield caused by increasing the amount of the chromium salt suggested that involvement of *gem*-dichromiomethane species was less likely in our metal-salt free system with **1a** compared with other chromium-catalyzed cyclopropanation developed by Takai *et al.*

**Scheme 3:**
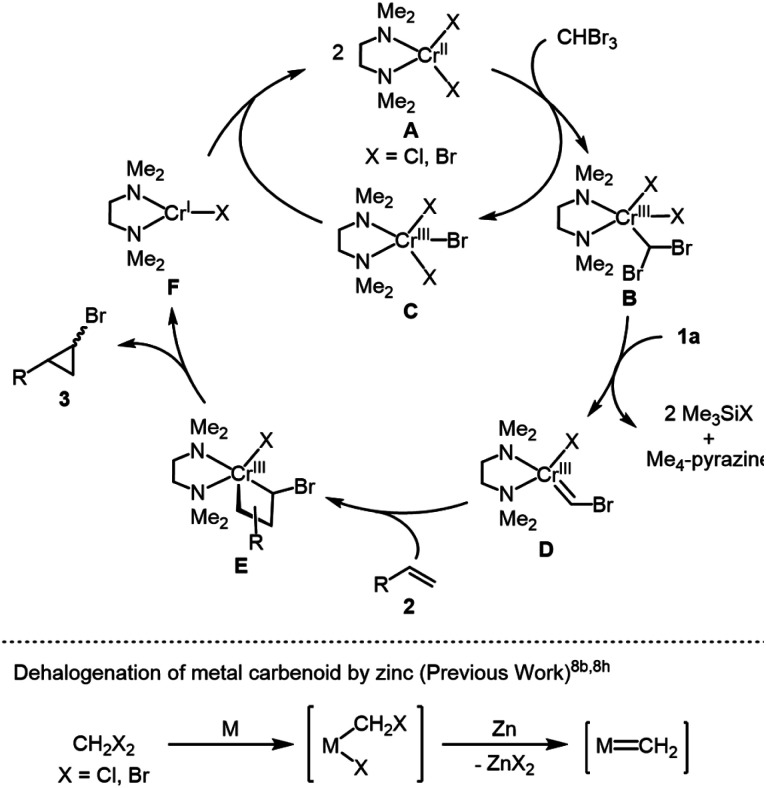
Proposed reaction mechanism.

On the basis of these findings, we propose the reaction mechanism shown in Scheme 3. The initial step is the activation of bromoform by chromium(ii) species **A** to form (dibromomethyl)chromium(iii) species **B** accompanied by the formation of an equimolar amount of chromium(iii) trihalide **C**, which can be reduced by **1a** or *in situ*-generated chromium(i) halide **F** (*vide infra*). Species **B** is dehalogenated by **1a** to afford (bromomethylidene)chromium(iii) **D** along with the elimination of Me_4_-pyrazine and 2 equiv. of Me_3_SiX (X = Cl, Br), whose reactivity is assumed due to the reductive dehalogenation of vicinal dihaloalkanes by the organosilicon-based reductant **1d** leading to the formation of alkenes.^12*a*^ In addition, the generation of metal carbene species by the dehalogenation of metal carbenoids with zinc powder was proposed for nickel- or cobalt-catalyzed cyclopropanation of alkenes with dibromomethane or dichloromethane (Scheme 3).^8*b*,8*h*^ Finally, the reaction of **D** with alkenes gives 4-membered metallacycle **E**, whose reductive elimination affords the desired bromocyclopropane together with a low valent nascent chromium(i) species **F**. The resulting **F** reacts with chromium(iii) trihalide **C** to regenerate chromium(ii) species **A** through comproportionation. Accordingly, **1a** has dual functions to reduce not only a catalyst precursor, CrCl_3_(tmeda), at the initial step, but also mainly the chromium(iii) species **B** for generating mononuclear chromium carbene species **D** as a key intermediate.

## Conclusions

In summary, we developed chromium-catalyzed bromocyclopropanation of alkenes with bromoform using an organosilicon-based reductant **1a**. The desired bromocyclopropanes were obtained in moderate to high yields with good *trans* selectivity, and the reaction was applicable to allyl ether derivatives, allyl carbonate, allylamine, and simple α-olefins. Control experiments suggested that **1a** played an important role in reducing the (dibromomethyl)chromium(iii) species to generate mononuclear (bromomethylidene)chromium(iii) as a key intermediate. Further exploration to discover the unique metal salt-free reductive transformation of organic compounds is ongoing in our laboratory.

## Conflicts of interest

The author declares no conflict of interest.

## Supplementary Material

SC-011-D0SC00964D-s001

SC-011-D0SC00964D-s002
