# Supplementary material for: Chromium-catalyzed cyclopropanation of alkenes with bromoform in the presence of 2,3,5,6-tetramethyl-1,4-bis(trimethylsilyl)-1,4-dihydropyrazine
Source: Chem Sci. 2020 Mar 11;11(14):3604–9. doi: 10.1039/d0sc00964d (PMC8152687; doi:10.1039/d0sc00964d)
Supplement: SC-011-D0SC00964D-s001 [file SC-011-D0SC00964D-s001.pdf]

Electronic Supplementary Material (ESI) for Chemical Science.
This journal is © The Royal Society of Chemistry 2020

Supporting Information

for

Chromium-Catalyzed Cyclopropanation of Alkenes with Bromoform

in the Presence of 2,3,5,6-Tetramethyl-1,4-bis(trimethylsilyl)-1,4-dihydropyrazine

Hideaki Ikeda, Kohei Nishi, Hayato Tsurugi,* and Kazushi Mashima*

Department of Chemistry, Graduate School of Engineering Science,

Osaka University, Toyonaka, Osaka 560-8531, Japan

E-mail: mashima@chem.es.osaka-u.ac.jp; tsurugi@chem.es.osaka-u.ac.jp

Contents

1. **General Information for Experimental Details**……………………………….…..…...S3

2. **Optimization Study for Chromium-catalyzed Cyclopropanation**………..……..S4

3. **Substrate Limitations of Alkenes and Halomethanes**…….……….…………….S6

4. **General Procedure for Chromium-catalyzed Cyclopropanation**………………S6

5. **Kinetic Study**………...………………………………………………………...S19

6. **Control Experiments**……………….…………………………………………S21

7. **X-ray Diffraction Analysis**…………………….…………………………...S24

8. **References**…………….…………………………………………………...S26

9. **NMR Spectra**………………..……………………………………………...S27

1. General Information for Experimental Details

All manipulations involving air- and moisture-sensitive organometallic compounds were carried out under argon using the standard Schlenk technique or argon-filled glovebox. Hexane, THF, and toluene were dried and deoxygenated by using Grubbs column (Glass Counter Solvent Dispending System, Nikko Hansen & Co., Ltd.). Bromoform, dibromochloromethane, bromodichloromethane, THF-d_8, and 1,2-dimethoxyethane (DME) were distilled over CaH_2 before use. $CrCl_3(thf)_3$, $CrCl_2$, Mn powder, Zn powder, $MnCl_2$, $ZnCl_2$, group 4-6 and late transition metal salts used in Table S1, tetrakis(dimethylamino)ethylene (TDAE), NaI, and nitrogen- and phosphine-based ligands (TMEDA, **L2-L15**) were purchased and used as recieved. $CrCl_3$(tmeda),[1] $[CrCl_2(tmeda)]_2$,[1] **L1**,[2] (diiodomethyl)trimethylsilane,[3] Takai's dichromium complex (**Cr_2-$SiMe_3$**),[1] 1,4-bis(trimethylsilyl)-1,4-dihydropyrazine derivatives (**1a**-**1c**), and 1,1'-bis(trimethylsilyl)-4,4'-bipyridinylidene (**1d**)[4] were prepared according to the literature procedures. Alkenes (**2a**, **2b**, **2h**-**2j**, **2l**-**2u**) were purchased, and allyl aryl ethers (**2c**-**2g**) and allyl(dibenzyl)amine (**2k**) were prepared according to the literature procedures.[5] ^{1}H NMR (400 MHz) and $^{13}C\{^1H\}$ NMR (100 MHz) spectra were measured on BRUKER AVANCEIII-400 spectrometers. ^{1}H and $^{13}C\{^1H\}$ NMR chemical shifts were reported in ppm and referenced to residual proton signal of the solvent (^{1}H: δ = 7.26, 1.94, and 1.72 ppm for $CDCl_3$, CD_3CN, and THF-d_8, respectively) or the solvent itself ($^{13}C\{^1H\}$: δ = 77.16 ppm for $CDCl_3$). High resolution mass spectra were recorded on a JEOL JMS-700. GC analyses were recorded on Shimadzu GC-2014 gas chromatograph with J&W Scientific DB-5 column. GC-MS analyses were performed with Shimadzu GCMS-QP2010 Plus spectrometer with Shimadzu GC-2010 equipped with J&W Scientific DB-1 column. IR spectra were recorded on JASCO FT/IR-410 and JASCO FT/IR-4000. Melting points were measured on BUCHI Melting Point M-565.

2. Optimization Study for Chromium-catalyzed Cyclopropanation

Table S1. Screening of Transition Metal Catalysts

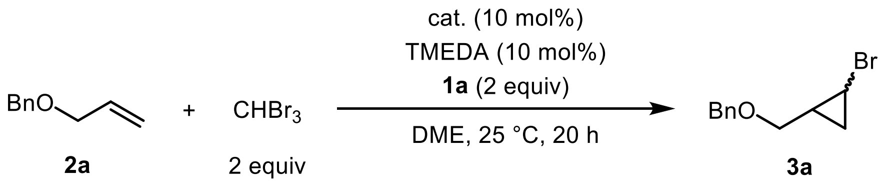

Entry	cat.	Yield (%)[a]	*trans* : *cis*[a]
1	$TiCl_3(thf)_3$	0	N/A
2	$CpTiCl_3$	0	N/A
3	$ZrCl_4$	0	N/A
4	$HfCl_4$	0	N/A
5	VCl_3	0	N/A
6	$NbCl_5$	0	N/A
7	$TaCl_5$	0	N/A
8[b]	$CrCl_3(thf)_3$	81	90 : 10
9[c]	$CrCl_2$	65	92 : 8
10	$Cr(acac)_3$	0	N/A
11	$Cr(OAc)_3$	0	N/A
12	$MoCl_3(thf)_3$	0	N/A
13	WCl_4	0	N/A
14	$CoCl_2$	0	N/A
15	$Co(acac)_2$	0	N/A
16	$Rh_2(OAc)_4$	0	N/A
17	$NiCl_2$	0	N/A
18	$Ni(acac)_2$	0	N/A
19	$ZnCl_2$	0	N/A

[a]Determined by ^{1}H NMR analysis using 1,3,5-trimethoxybenzene as an internal standard. [b]24 h. [c]THF, 24 h.

Table S2. Screening of Ligands

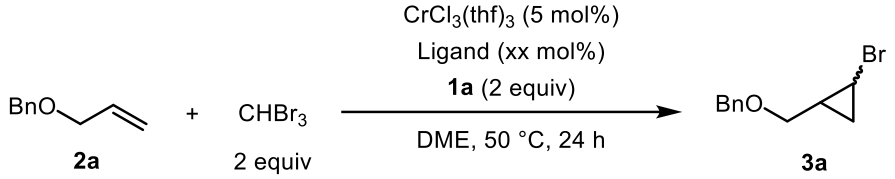

Entry	Ligand (mol%)	Yield (%)[a]	*trans* : *cis*[a]
1	TMEDA (5)	98	89 : 11
2[b]	**L1** (5)	97	90 : 10
3[c,d]	**L1** (10)	92	93 : 7
4	**L7** (10)	5	60 : 40
5	**L8** (10)	0	N/A
6	**L9** (5)	0	N/A
7	**L10** (5)	0	N/A
8	**L11** (5)	12	58 : 42
9	**L12** (5)	8	63 : 37
10	**L13** (10)	9	56 : 44
11	**L14** (10)	0	N/A
12	**L15** (10)	27	59 : 41
13[e]	-	8	63 : 37

[a]Determined by ^{1}H NMR analysis using 1,3,5-trimethoxybenzene as an internal standard. [b]*trans/cis* mixture of **L1** was used. [c](R,R)-**L1** was used. [d]$CrCl_3(thf)_3$ (10mol%), r.t., 36 h. [e]Under CO atmosphere (1 atm).

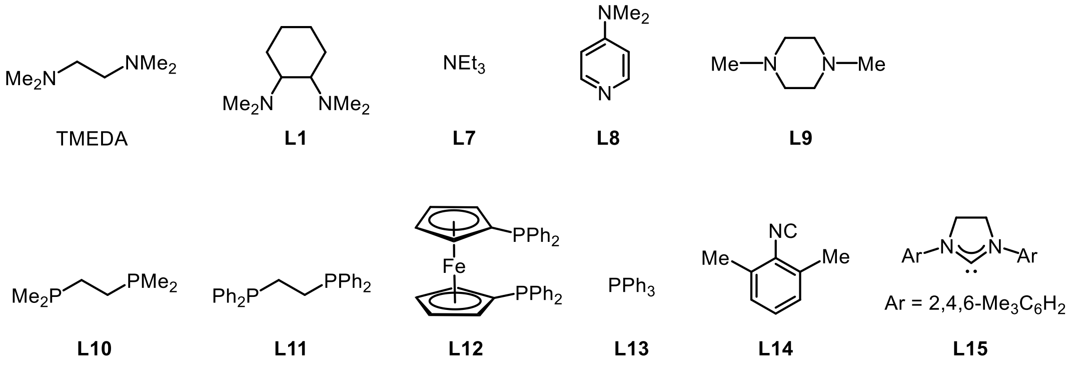

3. Substrate Limitations of Alkenes and Halomethanes

No cyclopropanation reaction or quite low yield of corresponding cyclopropanes were observed when following substrates were used for the chromium-catalyzed cyclopropanation under the optimized condition.

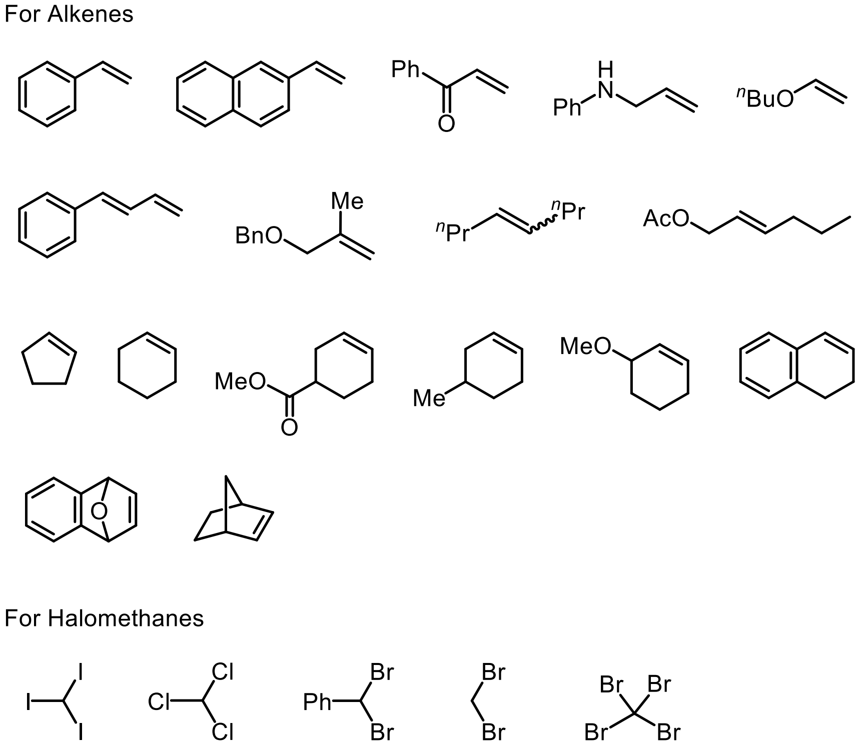

4. General Procedure for Chromium-catalyzed Cyclopropanation

For Optimization Study (Table 1) and Cyclopropanation Using Several Trihalomethanes (Scheme 1)

To a solution of $CrCl_3(thf)_3$ (5-10 mol%), reductant (0.20-0.60 mmol, 2-6 equiv), and ligand (5-10 mol%) in 1,2-dimethoxyethane (1.0 mL) was added **2a** (0.10 mmol) and the corresponding trihalomethane (0.20 mmol, 2 equiv). The reaction mixture was warmed up to noted temperature, and then stirred for 24 hours. The reaction was quenched by adding water and brine, followed by the addition of 1,3,5-trimethoxybenzene as an internal standard. Organic compounds were extracted with EtOAc (2-3 mL), then the

solvent was removed under reduced pressure. The yield and ratio of isomers for crude products were determined by ^{1}H NMR analysis.

For Scope of Substrates (Table 2)

To a solution of $CrCl_3(thf)_3$ (5-10 mol%), **1a** (226 mg, 0.80 mmol, 2 equiv), and TMEDA (5-10 mol%) in 1,2-dimethoxyethane (4.0 mL) was added alkene (0.40 mmol) and $CHBr_3$ (70.0 μL, 0.80 mmol, 2 equiv). The reaction mixture was warmed up to 50 °C, and then stirred for 24 hours. After quenching the reaction mixture by adding water and brine, organic compounds were extracted with EtOAc (5 mL x 4). The combined organic extracts were dried over Na_2SO_4, filtered, and the solvent was removed under reduced pressure. The crude product was purified by flash column chromatography to give bromocyclopropanes **3**.

Data of Cyclopropane Products

(2-Bromocyclopropyl)methyl benzyl ether (**3a**)

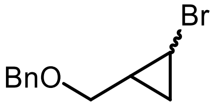

Isolated as colorless oil (89.9 mg, 93% yield, *trans* : *cis* = 89 : 11).
^{1}H NMR ($CDCl_3$, 400 MHz, 303 K):
trans-**3a**; δ 7.39-7.27 (m, 5H, Ph), 4.53 (d, *J* = 2.2 Hz, 2H, PhC*H*$_2$-), 3.47 (dd, *J* = 6.0, 10.5 Hz, 1H, PhCH$_2$OC*H*H-), 3.36 (dd, *J* = 6.6, 10.5 Hz, 1H, PhCH$_2$OCH*H*-), 2.78 (dt, *J* = 3.6, 7.2 Hz, 1H, CHBr), 1.60-1.56 (m, 1H, cyclopropyl), 1.11-1.06 (m, 1H, cyclopropyl), 1.01-0.96 (m, 1H, cyclopropyl) ppm.
cis-**3a**; δ 7.40-7.27 (m, 5H, Ph), 4.58 (d, *J* = 1.5 Hz, 2H, PhC*H*$_2$-), 3.72-3.68 (m, 1H, PhCH$_2$OC*H*H-), 3.61-3.56 (m, 1H, PhCH$_2$OCH*H*-), 3.12 (dt, *J* = 4.4, 7.3 Hz, 1H, CHBr), 1.30-1.25 (m, 2H, cyclopropyl), 0.71-0.70 (m, 1H, cyclopropyl) ppm.
^{13}C{^{1}H} NMR ($CDCl_3$, 100 MHz, 303 K):
trans-**3a**; δ 138.2, 128.6, 127.8, 127.7, 72.8, 71.0, 22.4, 17.9, 14.1 ppm.
cis-**3a**; δ 138.4, 128.5, 128.0, 127.8, 73.1, 71.7, 21.6, 16.6, 13.5 ppm.
IR (KBr): ν_{max} = 2963 (br), 1599 (m), 1496 (m), 1261 (s), 1094 (br), 1032 (br), 796 (s),

753 (m), 690 (m) cm^{-1}.

HRMS (EI): *m/z* calcd. for $[C_{11}H_{13}BrO]^+$ 240.0150; found 240.0159.

(2-Bromocyclopropyl)methyl phenyl ether (**3b**)

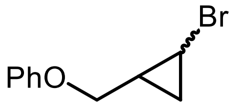

Isolated as colorless oil (83.1 mg, 92% yield, *trans* : *cis* = 90 : 10).

^{1}H NMR ($CDCl_3$, 400 MHz, 303 K):

trans-**3b**; δ 7.30-7.25 (m, 2H, Ph), 6.98-6.94 (m, 1H, Ph), 6.90-6.87 (m, 2H, Ph), 3.95 (dd, *J* = 6.0, 10.2 Hz, 1H, PhOC*H*H-), 3.90 (dd, *J* = 6.3, 10.2 Hz, 1H, PhOCH*H*-), 2.90 (dt, *J* = 3.8, 7.6 Hz, 1H, CHBr), 1.76-1.72 (m, 1H, cyclopropyl), 1.20-1.12 (m, 2H, cyclopropyl) ppm.

cis-**3b**; δ 7.30-7.25 (m, 2H, Ph), 6.98-6.94 (m, 1H, Ph), 6.90-6.87 (m, 2H, Ph), 4.16 (dd, *J* = 6.1, 10.2 Hz, 1H, PhOC*H*H-), 4.11 (dd, *J* = 7.5, 10.2 Hz, 1H, PhOCH*H*-), 3.18 (dt, *J* = 4.5, 7.3 Hz, 1H, CHBr), 1.47-1.44 (m, 1H, cyclopropyl), 1.39-1.33 (m, 1H, cyclopropyl), 0.66-0.63 (m, 1H, cyclopropyl) ppm.

^{13}C{^{1}H} NMR ($CDCl_3$, 100 MHz, 303 K):

trans-**3b**; δ 158.7, 129.6, 121.2, 114.8, 68.8, 21.9, 17.4, 14.2 ppm.

cis-**3b**; δ 158.7, 129.6, 121.0, 114.9, 69.7, 21.1, 16.1, 13.7 ppm.

IR (KBr): ν_{max} = 2916 (br), 1598 (m), 1496 (m), 1242 (br), 1033 (m), 752 (s), 691 (m) cm^{-1}.

HRMS (EI): *m/z* calcd. for $[C_{10}H_{11}BrO]^+$ 225.9993; found 225.9993.

(2-Bromocyclopropyl)methyl (4-cyanophenyl) ether (**3c**)

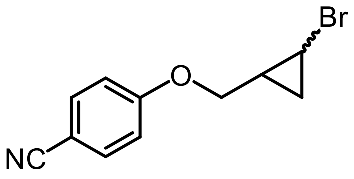

Isolated as white solid (63.7 mg, 64% yield, *trans* : *cis* = 90 : 10).

^{1}H NMR ($CDCl_3$, 400 MHz, 303 K):

trans-**3c**; δ 7.60-7.56 (m, 2H, Ar), 6.93-6.90 (m, 2H, Ar), 4.00 (dd, *J* = 6.1, 10.2 Hz, 1H, ArOC*H*H-), 3.91 (dd, *J* = 6.5, 10.2 Hz, 1H, ArOCH*H*-), 2.89 (dt, *J* = 3.8, 7.6 Hz, 1H, CHBr), 1.79-1.71 (m, 1H, cyclopropyl), 1.25-1.19 (m, 1H,

cyclopropyl), 1.16-1.11 (m,1H, cyclopropyl) ppm.

cis-**3c**; δ 7.60-7.56 (m, 2H, Ar), 7.00-6.97 (m, 2H, Ar), 4.22 (dd, *J* = 5.8, 10.0 Hz, 1H, ArOC*H*H-), 4.13 (dd, *J* = 7.7, 10.0 Hz, 1H, ArOCH*H*-), 3.19 (dt, *J* = 4.6, 7.2 Hz, 1H, CHBr), 1.46-1.36 (m, 2H, cyclopropyl), 0.89-0.84 (m, 1H, cyclopropyl) ppm.

$^{13}C\{^{1}H\}$ NMR ($CDCl_3$, 100 MHz, 303 K):

trans-**3c**; δ 161.9, 134.2, 119.2, 115.5, 104.6, 69.3, 21.5, 17.0, 14.3 ppm.

cis-**3c**; δ 161.9, 134.2, 119.2, 115.4, 104.6, 70.0, 20.8, 15.7, 13.7 ppm.

IR (KBr): ν_{max} = 2922 (br), 2221 (m), 1606 (m), 1507 (m), 1255 (s), 1175 (m), 1011 (m), 832 (m), 711 (m), 547 (m) cm^{-1}.

mp: 74 °C

HRMS (ESI): *m/z* calcd. for $[C_{11}H_{10}BrNO]^+$ ($[M+Na]^+$) 273.9838; found 273.9837.

(2-Bromocyclopropyl)methyl (4-chlorophenyl) ether (3d)

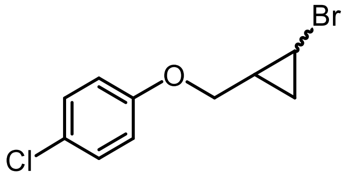

Isolated as colorless oil (75.8 mg, 72% yield, *trans* : *cis* = 90 : 10).

^{1}H NMR ($CDCl_3$, 400 MHz, 303 K):

trans-**3d**; δ 7.25-7.20 (m, 2H, Ar), 6.82-6.78 (m, 2H, Ar), 3.92 (dd, *J* = 6.0, 10.1 Hz, 1H, ArOC*H*H-), 3.85 (dd, *J* = 6.4, 10.1 Hz, 1H, ArOCH*H*-), 2.88 (dt, *J* = 4.0, 7.1 Hz, 1H, CHBr), 1.77-1.69 (m, 1H, cyclopropyl), 1.20-1.09 (m, 2H, cyclopropyl) ppm.

cis-**3d**; δ 7.25-7.20 (m, 2H, Ar), 6.88-6.86 (m, 2H, Ar), 4.13 (dd, *J* = 5.8, 10.0 Hz, 1H, ArOC*H*H-), 4.06 (dd, *J* = 7.6, 10.0 Hz, 1H, ArOCH*H*-), 3.18 (dt, *J* = 4.6, 7.3 Hz, 1H, CHBr), 1.44-1.35 (m, 2H, cyclopropyl), 0.87-0.82 (m, 1H, cyclopropyl) ppm.

$^{13}C\{^{1}H\}$ NMR ($CDCl_3$, 100 MHz, 303 K):

trans-**3d**; δ 157.3, 129.5, 126.2, 116.1, 69.3, 21.8, 17.3, 14.2 ppm.

cis-**3d**; δ 157.5, 129.5, 126.0, 116.2, 70.1, 21.0, 16.0, 13.7 ppm.

IR (KBr): ν_{max} = 2916 (br), 1493 (s), 1241 (br), 1092 (br), 1024 (br), 824 (s), 667 (m), 506 (m) cm^{-1}.

HRMS (EI): *m/z* calcd. for $[C_{10}H_{10}BrClO]^+$ 259.9604; found 259.9604.

(2-Bromocyclopropyl)methyl (4-bromophenyl) ether (3e)

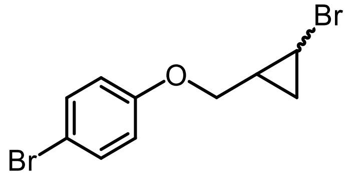

Isolated as colorless oil (106 mg, 87% yield, *trans* : *cis* = 88 : 12).

^{1}H NMR ($CDCl_3$, 400 MHz, 303 K):

trans-**3e**; δ 7.39-7.34 (m, 2H, Ar), 6.77-6.73 (m, 2H, Ar), 3.92 (dd, *J* = 6.0, 10.2 Hz, 1H, ArOC*H*H-), 3.84 (dd, *J* = 6.4, 10.2 Hz, 1H, ArOCH*H*-), 2.88 (dt, *J* = 4.0, 7.1 Hz, 1H, CHBr), 1.76-1.69 (m, 1H, cyclopropyl), 1.20-1.09 (m, 2H, cyclopropyl) ppm.

cis-**3e**; δ 7.39-7.34 (m, 2H, Ar), 6.84-6.79 (m, 2H, Ar), 4.13 (dd, *J* = 5.9, 10.0 Hz, 1H, ArOC*H*H-), 4.06 (dd, *J* = 7.6, 10.0 Hz, 1H, ArOCH*H*-), 3.18 (dt, *J* = 4.5, 7.3 Hz, 1H, CHBr), 1.49-1.33 (m, 1H, cyclopropyl), 0.86-0.82 (m, 1H, cyclopropyl), 0.65-0.63 (m, 1H, cyclopropyl) ppm.

$^{13}C\{^{1}H\}$ NMR ($CDCl_3$, 100 MHz, 303 K):

trans-**3e**; δ 157.8, 132.5, 116.6, 113.4, 69.2, 21.8, 17.3, 14.2 ppm.

cis-**3e**; δ 157.8, 132.4, 116.7, 113.4, 70.0, 21.0, 15.9, 13.7 ppm.

IR (KBr): ν_{max} = 2915 (br), 1590 (m), 1486 (s), 1242 (s), 1023 (m), 819 (m), 604 (m), 507 (m) cm^{-1}.

HRMS (EI): *m/z* calcd. for $[C_{10}H_{10}BrO]^+$ 303.9098; found 303.9098.

(2-Bromocyclopropyl)methyl (4-iodophenyl) ether (3f)

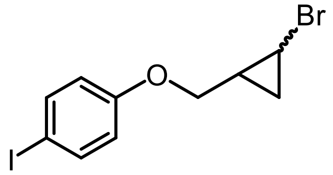

Isolated as colorless oil (90.1 mg, 64% yield, *trans* : *cis* = 87 : 13).

^{1}H NMR ($CDCl_3$, 400 MHz, 303 K):

trans-**3f**; δ 7.57-7.53 (m, 2H, Ph), 6.67-6.63 (m, 2H, Ph), 3.92 (dd, *J* = 6.0, 10.2 Hz, 1H, ArOC*H*H-), 3.84 (dd, *J* = 6.4, 10.2 Hz, 1H, ArOCH*H*-), 2.88 (dt, *J* = 4.0, 7.1 Hz, 1H, CHBr), 1.76-1.68 (m, 1H, cyclopropyl), 1.20-1.09 (m, 2H, cyclopropyl) ppm.

cis-**3f**; δ 7.57-7.53 (m, 2H, Ar), 6.73-6.68 (m, 2H, Ar), 4.13 (dd, *J* = 6.1, 10.0 Hz, 1H, ArOC*H*H-), 4.05 (dd, *J* = 7.6, 10.0 Hz, 1H, ArOCH*H*-), 3.18 (dt, *J* = 4.6, 7.4 Hz, 1H, CHBr), 1.43-1.33 (m, 1H, cyclopropyl), 0.89-0.82 (m, 2H, cyclopropyl) ppm.

$^{13}C\{^1H\}$ NMR ($CDCl_3$, 100 MHz, 303 K):

trans-**3f**; δ 158.6, 138.4, 117.2, 83.3, 69.0, 21.8, 17.3, 14.2 ppm.

cis-**3f**; δ 158.6, 138.4, 117.3, 83.1, 69.9, 21.0, 15.9, 13.7 ppm.

IR (KBr): ν_{max} = 2915 (br), 1585 (m), 1484 (s), 1240 (s), 1023 (m), 818 (m), 636 (w), 601 (w), 505 (w) cm^{-1}.

HRMS (EI): *m/z* calcd. for $[C_{10}H_{10}BrIO]^+$ 351.8960; found 351.8955.

(2-Bromocyclopropyl)methyl (4-methoxyphenyl) ether (3g)

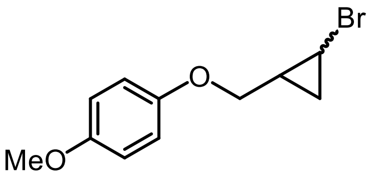

Isolated as colorless oil (71.2 mg, 69% yield, *trans* : *cis* = 94 : 6).

^1H NMR ($CDCl_3$, 400 MHz, 303 K):

trans-**3g**; δ 6.82 (m, 4H, Ar), 3.90 (dd, *J* = 6.0, 10.2 Hz, 1H, ArOC*H*H-), 3.83 (dd, *J* = 6.4, 10.2 Hz, 1H, ArOCH*H*-), 3.77 (s, 3H, MeO), 2.88 (dt, *J* = 3.8, 7.6 Hz, 1H, CHBr), 1.75-1.68 (m, 1H, cyclopropyl), 1.18-1.08 (m, 2H, cyclopropyl) ppm.

cis-**3g**; δ 6.91-6.82 (m, 4H, Ar), 4.11 (dd, *J* = 6.0, 10.1 Hz, 1H, ArOC*H*H-), 4.05 (dd, *J* = 7.6, 10.1 Hz, 1H, ArOCH*H*-), 3.17 (dt, *J* = 4.5, 7.3 Hz, 1H, CHBr), 1.45-1.26 (m, 2H, cyclopropyl), 0.85-0.81 (m, 1H, cyclopropyl) ppm.

$^{13}C\{^1H\}$ NMR ($CDCl_3$, 100 MHz, 303 K):

trans-**3g**; δ 154.3, 152.9, 116.0, 114.8, 69.8, 55.9, 22.1, 17.5, 14.2 ppm.

cis-**3g**; δ 154.3, 152.9, 116.0, 114.8, 70.6, 55.9, 22.1, 16.2, 13.7 ppm.

IR (KBr): ν_{max} = 2909 (br), 1508 (s), 1230 (s), 1038 (s), 825 (m), 743 (m), 614 (w) cm^{-1}.

HRMS (FAB): *m/z* calcd. for $[C_{11}H_{13}BrO_2]^+$ 256.0099; found 256.0095.

(2-Bromocyclopropyl)methyl butyl ether (3h)

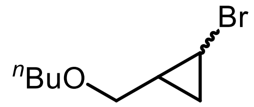

Isolated as colorless oil (67.2 mg, 81% yield, *trans* : *cis* = 87 : 13).

^1H NMR ($CDCl_3$, 400 MHz, 303 K):

trans-**3h**; δ 3.44-3.37 (m, 1+2H, ^{n}BuOC*H*H-/$CH_3(CH_2)_2CH_2O$-), 3.32 (dd, *J* = 6.4, 10.6 Hz, 1H, ^{n}BuOCH*H*-), 2.76 (dt, *J* = 3.8, 7.2 Hz, 1H, CHBr), 1.56-

1.51 (m, 4H, $CH_3(CH_2)_2CH_2O$-), 1.39-1.33 (m, 1H, cyclopropyl), 1.08-1.03 (m, 1H, cyclopropyl), 0.99-0.95 (m, 1H, cyclopropyl), 0.92 (t, *J* = 7.4 Hz, 3H, $CH_3(CH_2)_3O$-) ppm.

cis-**3h**; δ 3.61 (dd, *J* = 5.5, 10.6 Hz, 1H, ^{n}BuOC*H*H-), 3.54-3.46 (m, 2+1H, $CH_3(CH_2)_2CH_2O$-/^{n}BuOCH*H*-), 3.11 (dt, *J* = 4.4, 7.3 Hz, 1H, CHBr), 1.39-1.33 (m, 4H, $CH_3(CH_2)_2CH_2O$-), 0.99-0.91 (m, 3H, cyclopropyl), 0.93 (t, *J* = 7.3 Hz, 3H, $CH_3(CH_2)_3O$-) ppm.

$^{13}C\{^1H\}$ NMR ($CDCl_3$, 100 MHz, 303 K):

trans-**3h**; δ 71.3, 70.4, 31.6, 22.1, 19.2, 17.6, 13.7, 0.84 ppm.

cis-**3h**; δ 71.7, 70.6, 31.7, 21.4, 19.8, 16.3, 13.2, 1.21 ppm.

IR (KBr): ν_{max} = 2918 (br), 2850 (br), 1496 (m), 1261 (s), 1094 (br), 1024 (br), 798 (s) cm^{-1}.

HRMS (CI): *m/z* calcd. for $[C_8H_{15}BrO]^+$ ($[M+H]^+$) 207.0385; found 207.0380.

Benzoyl (2-bromocyclopropyl)methyl ether (3i)

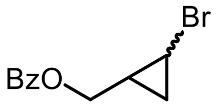

Isolated as colorless oil (76.0 mg, 75% yield, *trans* : *cis* = 89 : 11).

^1H NMR ($CDCl_3$, 400 MHz, 303 K):

trans-**3i**; δ 8.10-8.04 (m, 2H, Ph), 7.60-7.55 (m, 1H, Ph), 7.47-7.43 (m, 2H, Ph), 4.30 (dd, *J* = 6.5, 11.7 Hz,1H, BzOC*H*H-), 4.19 (dd, *J* = 7.3, 11.7 Hz, 1H, BzOCH*H*-), 2.91 (dt, *J* = 3.6, 7.5 Hz, 1H, CHBr), 1.80-1.72 (m, 1H, cyclopropyl), 1.21-1.09 (m, 2H, cyclopropyl) ppm.

cis-**3i**; δ 8.10-8.04 (m, 2H, Ph), 7.60-7.55 (m, 1H, Ph), 7.47-7.43 (m, 2H, Ph), 4.65 (dd, *J* = 6.0, 11.7 Hz, 1H, BzOC*H*H-), 4.33 (dd, *J* = 8.3, 11.7 Hz, 1H, BzOCH*H*-), 3.15 (dt, *J* = 4.6, 7.3 Hz, 1H, CHBr), 1.38-1.33 (m, 1H, cyclopropyl), 0.90-0.86 (m, 2H, cyclopropyl) ppm.

$^{13}C\{^1H\}$ NMR ($CDCl_3$, 100 MHz, 303 K):

trans-**3i**; δ 166.5, 133.2, 130.1, 129.8, 128.5, 66.1, 21.6, 17.4, 14.5 ppm.

cis-**3i**; δ 166.7, 133.1, 130.1, 129.8, 128.5, 66.6, 20.8, 15.6, 13.8 ppm.

IR (KBr): ν_{max} = 3063 (br), 2949 (br), 1719 (s), 1451 (m), 1273 (s), 1109 (s), 972 (w), 711 (s) cm^{-1}.

HRMS (ESI): *m/z* calcd. for $[C_{11}H_{11}BrO_2]^+$ ($[M+K]^+$) 292.9574; found 292.9572.

(2-Bromocyclopropyl)methyl methyl carbonate (3j)

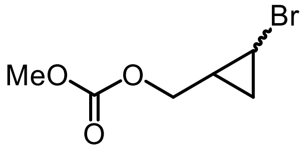

Isolated as pale yellow colored oil (50.2 mg, 60% yield, *trans* : *cis* = 81 : 19).

^{1}H NMR ($CDCl_3$, 400 MHz, 303 K):

trans-**3j**; δ 4.08 (dd, *J* = 6.7, 11.6 Hz, 1H, -OC*H*H-), 4.00 (dd, *J* = 7.4, 11.6 Hz, 1H, -OCH*H*-), 3.80 (s, 3H, Me), 2.83 (dt, *J* = 4.0, 7.5 Hz, 1H, CHBr), 1.70-1.63 (m, 1H, cyclopropyl), 1.19-1.12 (m, 1H, cyclopropyl), 1.07-1.02 (m, 1H, cyclopropyl) ppm.

cis-**3j**; δ 4.39 (dd, *J* = 6.0, 11.4 Hz, 1H, -OC*H*H-), 4.22 (dd, *J* = 7.9, 11.4 Hz, 1H, -OCH*H*-), 3.81 (s, 3H, Me), 3.11 (dt, *J* = 4.6, 7.3 Hz, 1H, CHBr), 1.37-1.28 (m, 1H, cyclopropyl), 0.84-0.77 (m, 2H, cyclopropyl) ppm.

^{13}C{^{1}H} NMR ($CDCl_3$, 100 MHz, 303 K):

trans-**3j**; δ 155.8, 69.3, 55.0, 21.3, 17.1, 14.4 ppm.

cis-**3j**; δ 155.8, 69.7, 55.0, 20.5, 15.4, 13.7 ppm.

IR (KBr): ν_{max} = 2957 (br), 1749 (s), 1444 (m), 1270 (s), 962 (m), 792 (m) cm^{-1}.

HRMS (EI): *m/z* calcd. for $[C_6H_9BrO_3]^+$ 207.9735; found 207.9741.

Dibenzyl (2-bromocyclopropyl)methyl amine (3k)

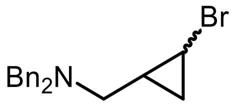

Isolated as colorless oil (84.4 mg, 64% yield, *trans* : *cis* = 84 : 16).

^{1}H NMR ($CDCl_3$, 400 MHz, 303 K):

trans-**3k**; δ 7.39 (d, *J* = 7.1 Hz, 4H, Ph), 7.32 (m, 4H, Ph), 7.26-7.22 (m, 2H, Ph), 3.72-3.59 (m, 4H, $(PhCH_2)_2NCH_2$-), 2.57-2.51 (m, 1+1 H, Bn_2NC*H*H-/CHBr), 2.25 (dd, *J* = 7.7, 13.6 Hz, 1H, Bn_2NCH*H*-), 1.49-1.41 (m, 1H, cyclopropyl), 1.04-0.99 (m, 1H, cyclopropyl), 0.72 (dd, *J* = 6.5, 13.6 Hz, 1H, cyclopropyl) ppm.

cis-**3k**; δ 7.40-7.37 (m, 4H, Ph), 7.33-7.29 (m, 4H, Ph), 7.26-7.22 (m, 2H, Ph), 3.72-3.59 (m, 4H, $(PhCH_2)_2NCH_2$-), 3.07-3.03 (m, 1H, CHBr), 2.78 (dd, *J* = 5.5, 13.0 Hz, 1H, Bn_2NC*H*H-), 2.61 (dd, *J* = 5.9, 13.0 Hz, 1H, Bn_2NCH*H*-), 1.26-1.18 (m, 1H, cyclopropyl), 1.12-1.08 (m, 1H, cyclopropyl), 0.55 (dd, *J* = 6.6, 11.2 Hz, 1H, cyclopropyl) ppm.

^{13}C{^{1}H} NMR ($CDCl_3$, 100 MHz, 303 K):

trans-**3k**; δ 139.7, 128.8, 128.4, 127.1, 58.3, 55.8, 20.3, 19.9, 14.9 ppm.

cis-**3k**; δ 139.8, 128.9, 128.3, 126.9, 57.9, 56.5, 20.3, 19.9, 14.4 ppm.

IR (KBr): ν_{max} = 3026 (w), 2801 (br), 1493 (m), 1451 (m), 1367 (m), 1239 (br), 1028 (w), 738 (s), 698 (s), 505 (w) cm^{-1}.

HRMS (EI): *m/z* calcd. for $[C_{18}H_{20}BrN]^+$ 329.0779; found 329.0779.

((2-bromocyclopropyl)methyl)benzene (3l)

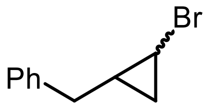

Isolated as colorless oil (58.3 mg, 69% yield, *trans* : *cis* = 86 : 14).

^{1}H NMR ($CDCl_3$, 400 MHz, 303 K):

trans-**3l**; δ 7.34-7.29 (m, 2H, Ph), 7.24-7.21 (m, 3H, Ph), 2.76-2.70 (m, 1H, CHBr), 2.75-2.70 (m, 1H, PhC*H*H-), 2.62 (dd, *J* = 7.1, 14.9 Hz, 1H, PhCH*H*-), 1.59-1.51 (m, 1H, cyclopropyl), 1.11-1.06 (m, 1H, cyclopropyl), 0.94-0.87 (m, 1H, cyclopropyl) ppm.

cis-**3l**; δ 7.34-7.29 (m, 2H, Ph), 7.24-7.21 (m, 3H, Ph), 3.17 (dt, *J* = 4.3, 7.4 Hz, 1H, CHBr), 2.95 (dd, *J* = 7.0, 15.0 Hz, 1H, PhC*H*H-), 2.79 (dd, *J* = 6.8, 15.0 Hz, 1H, PhCH*H*-), 1.32-1.27 (m, 1H, cyclopropyl), 1.22-1.16 (m, 1H, cyclopropyl), 0.74-0.71 (m, 1H, cyclopropyl) ppm.

$^{13}C\{^1H\}$ NMR ($CDCl_3$, 100 MHz, 303 K):

trans-**3l**; δ 139.9, 128.6, 128.5, 126.5, 38.3, 23.2, 19.8, 15.9 ppm.

cis-**3l**; δ 141.1, 128.6, 128.4, 126.3, 36.9, 23.8, 18.1, 15.3 ppm.

IR (KBr): ν_{max} = 3026 (br), 2917 (br), 1496 (m), 1452 (m), 1240 (m), 1032 (m), 735 (s), 698 (s) cm^{-1}.

HRMS (EI): *m/z* calcd. for $[C_{10}H_{11}Br]^+$ 210.0044; found 210.0046.

4-(2-bromocyclopropyl)butyl acetate (3m)

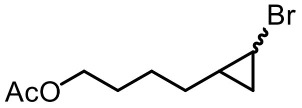

Isolated as colorless oil (69.3 mg, 74% yield, *trans* : *cis* = 84 : 16).

^{1}H NMR ($CDCl_3$, 400 MHz, 303 K):

trans-**3m**; δ 4.06 (t, *J* = 6.6 Hz, 2H, AcOC*H*$_2$-), 2.59 (dt, *J* = 3.2, 7.4 Hz, 1H, CHBr), 2.04 (s, 3H, AcO), 1.69-1.25 (m, 6H, AcOCH$_2$(C*H*$_2$)$_3$-), 1.23-1.14 (m, 1H, cyclopropyl), 1.02-

0.97 (m, 1H, cyclopropyl), 0.76 (dd, *J* = 6.3, 13.6 Hz, 1H, cyclopropyl) ppm.

cis-**3m**; δ 4.08 (t, *J* = 6.6 Hz, 2H, AcOCH_2-), 3.05 (dt, *J* = 4.2, 7.3 Hz, 1H, CHBr), 2.04 (s, 3H, AcO), 1.69-1.25 (m, 6H, AcOCH_2(CH_2)$_3$-), 0.87-0.81 (m, 1H, cyclopropyl), 0.68-0.61 (m, 1H, cyclopropyl), 0.53-0.49 (m, 1H, cyclopropyl) ppm.

^{13}C{^{1}H} NMR ($CDCl_3$, 100 MHz, 303 K):

trans-**3m**; δ 171.3, 64.5, 32.4, 28.4, 25.3, 22.8, 21.1, 19.6, 16.0 ppm.

cis-**3m**; δ 171.3, 64.6, 33.4, 30.9, 28.5, 25.5, 23.8, 16.8, 15.1 ppm.

IR (KBr): ν_{max} = 2962 (w), 1261 (s), 1097 (br), 1020 (br), 801 (s) cm^{-1}.

HRMS (CI): *m/z* calcd. for [$C_9H_{15}BrO_2$]$^+$ ([M+H]$^+$) 235.0333; found 235.0333.

1-Bromo-2-hexylcyclopropane (3n)

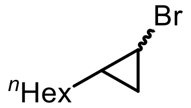

Isolated as colorless oil (60.4 mg, 74% yield, *trans* : *cis* = 83 : 17).

^{1}H NMR ($CDCl_3$, 400 MHz, 303 K):

trans-**3n**; δ 2.59 (dt, *J* = 3.0, 7.4 Hz, 1H, CHBr), 1.43-1.13 (m, 10+1H, CH_3(CH_2)$_5$-/cyclopropyl), 1.00-0.95 (m, 1H, cyclopropyl), 0.91-0.87 (m, 3H, CH_3(CH_2)$_5$-), 0.75 (dd, *J* = 6.2, 13.3 Hz, 1H, cyclopropyl) ppm.

cis-**3n**; δ 3.05 (dt, *J* = 4.2, 7.4 Hz, 1H, CHBr), 1.43-1.13 (m, 10+1H, CH_3(CH_2)$_5$-/cyclopropyl), 0.91-0.87 (m, 3H, CH_3(CH_2)$_5$-), 0.65-0.60 (m, 1H, cyclopropyl), 0.52-0.48 (m, 1H, cyclopropyl) ppm.

^{13}C{^{1}H} NMR ($CDCl_3$, 100 MHz, 303 K):

trans-**3n**; δ 32.9, 31.9, 29.1, 28.8, 23.1, 22.8, 20.2, 16.0, 14.2 ppm.

cis-**3n**; δ 32.0, 31.7, 31.3, 29.2, 24.2, 22.8, 20.9, 17.0, 15.1 ppm.

IR (KBr): ν_{max} = 2962 (w), 1261 (s), 1097 (br), 1020 (br), 801 (s) cm^{-1}.

HRMS (EI): *m/z* calcd. for [$C_9H_{17}Br$]$^+$ 204.0514; found 204.0519.

(2-bromocyclopropyl)cyclohexane (3o)

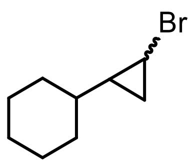

Isolated as colorless oil (42.3 mg, 52% yield, *trans* : *cis* = >99 : 1).

^{1}H NMR ($CDCl_3$, 400 MHz, 303 K):

trans-**3o**; δ 2.65 (dt, *J* = 3.6, 7.4 Hz, 1H, CHBr), 1.83-1.63 (m, 6H,

cyclohexyl), 1.26-1.05 (m, 5H, cyclohexyl), 0.96-0.91 (m, 1H, cyclopropyl), 0.82-0.77 (m, 1H, cyclopropyl), 0.67-0.62 (m, 1H, cyclopropyl) ppm.

$^{13}C\{^1H\}$ NMR ($CDCl_3$, 100 MHz, 303 K):

trans-**3o**; δ 41.6, 32.5, 32.0, 29.3, 26.5, 26.2, 26.2, 18.8, 14.9 ppm.

IR (KBr): ν_{max} = 2924 (s), 2853 (m), 1449 (m), 1261 (s), 1091 (br), 1019 (br), 805 (s), 700 (w) cm^{-1}.

HRMS (EI): *m/z* calcd. for $[C_9H_{15}Br]^+$ 202.0357; found 202.0355.

4-(2-bromocyclopropyl)-1-cyclohexene (3p)

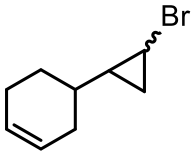

Isolated as colorless oil (46.2 mg, 64% yield, *trans* : *cis* = >99 : 1).

^1H NMR ($CDCl_3$, 400 MHz, 303 K):

trans-**3p**; δ 5.67-5.64 (m, 2H, -C*H*=C*H*-), 2.69 (dt, *J* = 3.5, 10.9 Hz, 1H, CHBr), 2.11-1.74 (m, 4H, -C*H*$_2$-CH=CH-C*H*$_2$-), 1.50-1.33 (m, 2H, -CH-C*H*$_2$-), 1.22-1.16 (m, 1H, -C*H*-cyclopropyl), 1.01-0.96 (m, 2H, cyclopropyl), 0.87-0.81 (m, 1H, cyclopropyl) ppm.

$^{13}C\{^1H\}$ NMR ($CDCl_3$, 100 MHz, 303 K):

trans-**3p**; δ 127.1, 125.9, 37.4, 30.8, 28.4, 27.9, 24.8, 18.6, 14.6 ppm.

IR (KBr): ν_{max} = 3432 (br), 2919 (w), 2345 (w), 1071 (br), 650 (w) cm^{-1}.

HRMS (EI): *m/z* calcd. for $[C_9H_{13}Br]^+$ ($[M-H]^+$) 199.0122; found 199.0120.

1-bromo-2-(3-methyl-3-buten-1-yl)cyclopropane (3q)

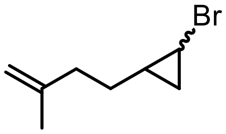

Isolated as colorless oil (54.2 mg, 72% yield, *trans* : *cis* = 69 : 31).

^1H NMR ($CDCl_3$, 400 MHz, 303 K):

trans-**3q**; δ 4.73-4.69 (m, 2H, (Me)(R)C=C*H*$_2$), 2.61 (dt, *J* = 3.5, 7.4 Hz, 1H, CHBr), 2.14-2.10 (m, 2H, -(C*H*$_2$)$_2$-), 1.72 (s, 3H, Me) 1.46-1.38 (m, 2H, -(C*H*$_2$)$_2$-), 1.28-1.15 (m, 1H, cyclopropyl), 1.02-0.97 (m, 1H, cyclopropyl), 0.80-0.75 (m, 1H, cyclopropyl) ppm.

cis-**3q**; δ 4.73-4.69 (m, 2H, (Me)(R)C=C*H*$_2$), 3.10-3.04 (m, 1H, CHBr), 2.14-2.10 (m, 2H, -(C*H*$_2$)$_2$-), 1.75 (s, 3H, Me) 1.46-1.38 (m, 2H, -(C*H*$_2$)$_2$-), 1.28-1.15 (m, 1H, cyclopropyl),

0.90-0.84 (m, 1H, cyclopropyl), 0.67-0.54 (m, 1H, cyclopropyl) ppm.

$^{13}C\{^{1}H\}$ NMR ($CDCl_3$, 100 MHz, 303 K):

trans-**3q**; δ 145.2, 110.5, 36.9, 31.2, 22.8, 22.6, 19.9, 16.1 ppm.

cis-**3q**; δ 145.2 110.2, 37.4, 29.5, 22.6, 22.1, 20.6, 13.7 ppm.

IR (KBr): ν_{max} = 3434 (br), 2925 (m), 2370 (w), 1449 (w), 1079 (br), 888 (w) cm^{-1}.

HRMS (EI): *m/z* calcd. for $[C_8H_{13}Br]^+$ ($[M-H]^+$) 187.0122; found 187.0116.

(3-bromocyclopropane-1,2-diyl)bis(methylene) diacetate (3r)

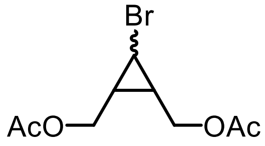

47% yield, *trans* : *cis* = >99 : 1 (determined by ^{1}H NMR analysis using 1,3,5-trimethoxybenzene as an internal standard).

trans-**3r**; δ 4.23 (dd, *J* = 7.2, 12.1 Hz, 2H, AcOCH_2-), 4.03 (dd, *J* = 7.2, 12.1 Hz, 2H, AcOCH_2-), 2.82 (t, *J* = 4.1 Hz, 1H, CHBr), 1.82-1.77 (m, 2H, cyclopropyl), 2.07 (s, 3H, Me) ppm.

8-bromobicyclo[5.1.0]nonane (3s)

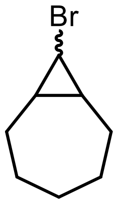

Isolated as colorless oil (53.9 mg, 71% yield, *trans* : *cis* = >99 : 1).

^{1}H NMR ($CDCl_3$, 400 MHz, 303 K):

trans-**3s**; δ 2.60 (t, *J* = 3.4 Hz, 1H, CHBr), 2.27-2.21 (m, 2H, -(C*H*)$_2$CHBr), 1.79-1.65 (m, 3H, -CH_2-), 1.39-1.29 (m, 4H, -CH_2-), 1.20-1.13 (m, 1H, -CH_2-), 1.05-0.96 (m, 2H, -CH_2-) ppm.

$^{13}C\{^{1}H\}$ NMR ($CDCl_3$, 100 MHz, 303 K):

trans-**3s**; δ 32.6, 29.9, 29.4, 27.9, 27.5 ppm.

IR (KBr): ν_{max} = 3443 (br), 2920 (m), 2369 (w), 1092 (br), 800 (w) cm^{-1}.

HRMS (EI): *m/z* calcd. for $[C_8H_{13}Br]^+$ 188.0201; found 188.0202.

9-bromobicyclo[6.1.0]nonane (3t)

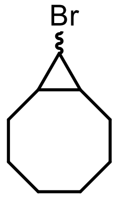

Isolated as colorless oil (64.5 mg, 79% yield, *trans* : *cis* = >99 : 1).

^{1}H NMR ($CDCl_3$, 400 MHz, 303 K):

trans-**3t**; δ 2.32 (t, *J* = 3.6 Hz, 1H, CHBr), 2.18-2.13 (m, 2H, -(C*H*)$_2$CHBr), 1.63-1.52 (m, 4H, -CH_2-), 1.43-1.29 (m, 4H, -CH_2-), 1.18-1.14 (m, 2H, -CH_2-), 0.95-0.86 (m, 2H, -CH_2-) ppm.

^{13}C{^{1}H} NMR ($CDCl_3$, 100 MHz, 303 K):

trans-**3t**; δ 28.9, 27.1, 26.4, 25.7, 24.5 ppm.

IR (KBr): ν_{max} = 2963 (m), 1261 (s), 1097 (br), 1019 (br), 795 (s), 699 (w) cm^{-1}.

HRMS (EI): *m/z* calcd. for $[C_9H_{15}Br]^+$ 202.0357; found 202.0360.

6b,7a-dihydro-7*H*-cyclopropa[*a*]acenaphthylene (3u)

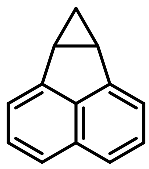

Isolated as pale brown solid (23.2 mg, 35% yield).

^{1}H NMR ($CDCl_3$, 400 MHz, 303 K):

δ 7.56-7.54 (m, 2H, Ar), 7.40-7.36 (m, 4H, Ar), 3.02 (dd, *J* = 3.7, 7.9 Hz, 2H, cyclopropyl), 1.54-1.14 (m, 1H, cyclopropyl), 0.79-0.76 (m, 1H, cyclopropyl) ppm.

^{13}C{^{1}H} NMR ($CDCl_3$, 100 MHz, 303 K):

δ 146.4, 136.5, 132.0, 127.3, 122.7, 119.4, 27.0, 23.9 ppm.

IR (KBr): ν_{max} = 3434 (br), 3036 (w), 1606 (m), 1363 (w), 1262 (w), 1035 (m), 821 (s), 783 (s), 768 (s) cm^{-1}.

HRMS (EI): *m/z* calcd. for $[C_{13}H_{10}]^+$ 166.0783; found 166.0776.

5. Kinetic Study

Normalized Time Scale Analysis for the Catalyst $CrCl_3(thf)_3$/TMEDA

We determined a power value on the concentration of the catalyst $CrCl_3(thf)_3$/TMEDA by using a normalized time scale analysis.[6] A mixture of $CrCl_3(thf)_3$ (0.005 M), TMEDA (0.005 M), **1a** (226 mg, 0.80 mmol, 0.20 M), allyl benzyl ether (**2a**, 61.7 μL, 0.40 mmol, 0.10 M), and bromoform (70.0 μL, 0.80 mmol, 0.20 M) in 1,2-dimethoxyethane (4.0 mL) with pentadecane as an internal standard was stirred at 50 °C. A small portion of the reaction mixture was sampled by using a syringe, and then quenched by sat. NaCl aq for measuring the yield of **3a** by GC analysis. The same operations were conducted for the reaction mixture containing $CrCl_3(thf)_3$ (0.004 M and 0.010 M). As shown in Figure S1, the concentration of **3a** was plotted against a normalized time scale, $t[\text{cat.}]T^n$ ($[\text{cat.}]T$ = concentration of total amount of the catalyst), and we adjusted the power value, n, until all the corrected yield curves overlay. In this case, we finally determined that the value of n was 0 (Figure S1). Moreover, the induction period was not observed in the early stage of the reaction even at low catalyst loadings.

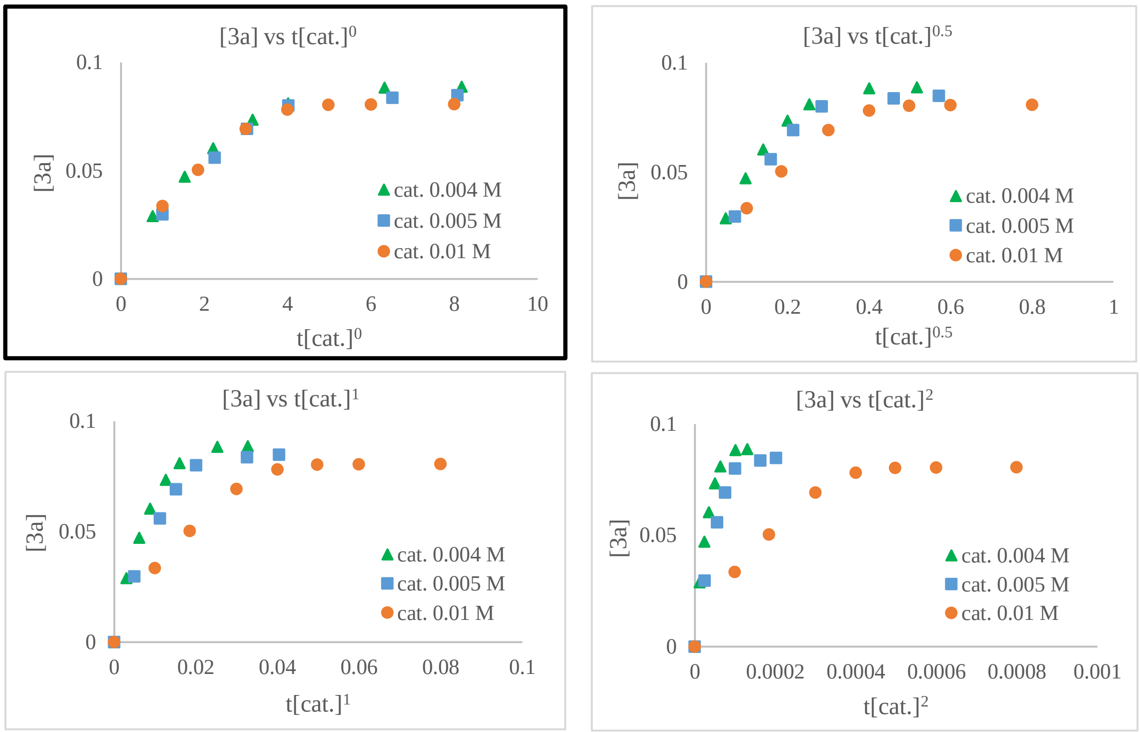

Figure S1. Normalized time scale analysis of Cr-catalyzed cyclopropanation

Normalized Time Scale Analysis for Allyl Benzyl Ether (2a)

We determined a power value on the concentration of the catalyst $CrCl_3(thf)_3$/TMEDA by using a normalized time scale analysis.[6] A mixture of $CrCl_3(thf)_3$ (0.005 M), TMEDA (0.005 M), **1a** (226 mg, 0.80 mmol, 0.20 M), allyl benzyl ether (**2a**, 61.7 μL, 0.40 mmol, 0.10 M), and bromoform (70.0 μL, 0.80 mmol, 0.20 M) in 1,2-dimethoxyethane (4.0 mL) with pentadecane as an internal standard was stirred at 50 °C. A small portion of the reaction mixture was sampled by using a syringe, and then quenched by sat. NaCl aq for measuring the yield of **3a** by GC analysis. The same operations were conducted for the reaction mixture containing **2a** (0.080 M and 0.120 M). As shown in Figure S2, the concentration of **3a** was plotted against a normalized time scale, $\Sigma[2a]^a\Delta t$, and we adjusted the power value, *a*, until all the corrected yield curves overlay. In this case, we finally determined that the value of *a* was 0 (Figure S2).

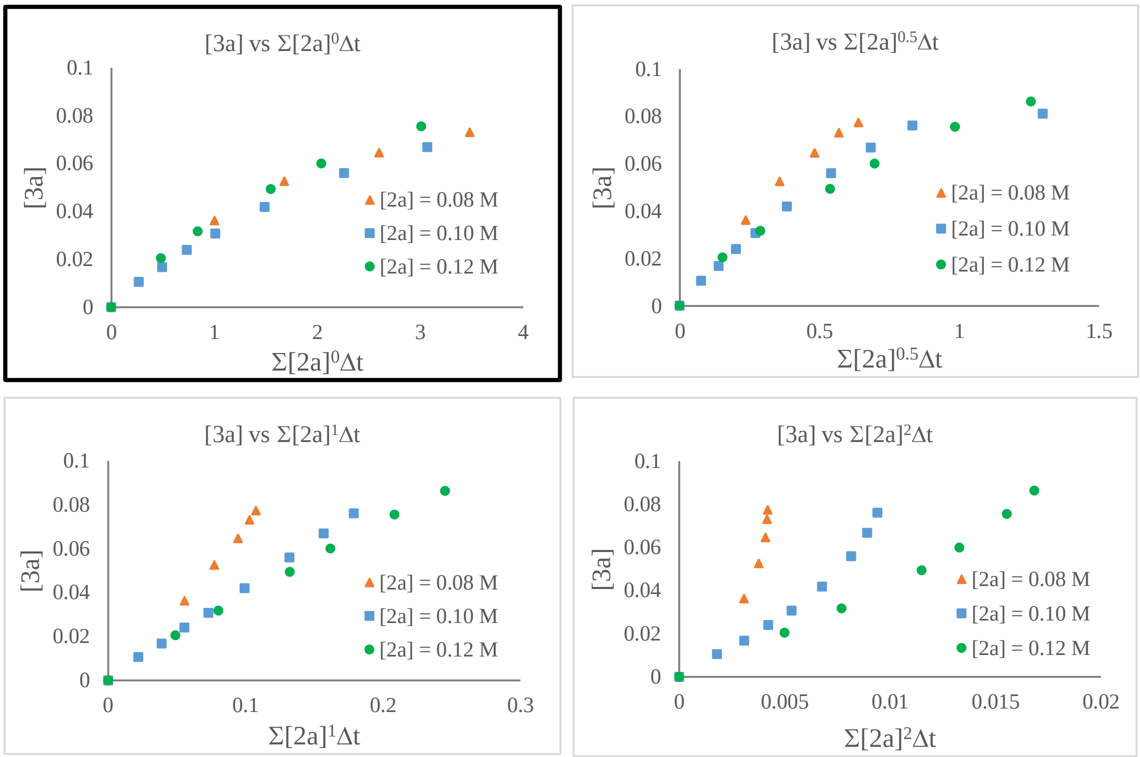

Figure S2. Normalized time scale analysis of Cr-catalyzed cyclopropanation to determine the order in allyl benzyl ether (**2a**)

6. Control Experiments

Trials for Synthesis of Bromomethyl-bridged Dichromium Complex

To a suspension of $CrCl_2$ (498 mg, 4.05 mmol, 4 equiv) in THF (6 mL) was added a THF (3 mL) solution of $CHBr_3$ (87.6 μL, 1.00 mmol) at -40 °C. The reaction mixture was slowly warmed up to room temperature with stirring. After 30 minutes, the color of reaction mixture changed to deep red. The reaction mixture was stirred at room temperature for 2 hours, and then deep red solution was transferred to another Schlenk through filtration. The solvent was removed under reduced pressure to give dark red solid (297 mg, similar color compared with dichromium complexes reported by Takai[1a] and Anwander[1b]). ^{1}H NMR spectrum was not observed because of paramagnetic property of chromium. Recrystallization in THF at -20 °C afforded red microcrystals; however, the quality of obtained crystal was not enough for X-ray diffraction analysis. In addition, the immediate color change from red to green was observed upon exposure to air, suggesting that obtained product was quite unstable.

In addition, stoichiometric cyclopropanation of **2a** with bromoform using excess amount of $CrCl_2$/TMEDA did not proceed to give bromocyclopropane **3a** (eq. S1), suggesting that bromomethyl-bridged dichromium complex did not serve a reactive chromium carbene complex even if the formation of dichromium complexes occured.

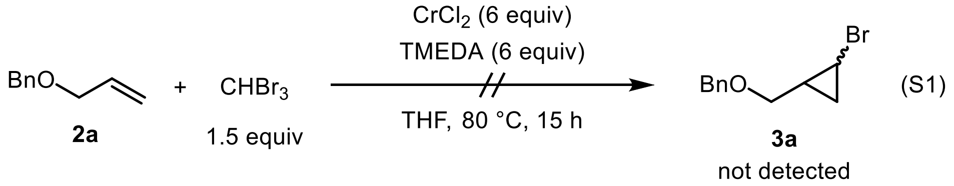

Reactivity of (Trimethylsilyl)methyl-bridged Dichromium Complex **Cr_2-SiMe$_3$**

We checked the reactivity of isolated dichromium complex **Cr_2-SiMe$_3$** combined with organosilicon reductant **1a**. At first, the reduction of **Cr_2-SiMe$_3$** was not observed although it was judged from that color change did not occurred (eq. S2). Moreover, stoichiometric cyclopropanation by **Cr_2-SiMe$_3$** with **1a** was attempted; however, no acceleration effect of **1a** was observed (eq. S3), indicating that the reaction of *in situ*-generated dichromium complex, such as **Cr_2-SiMe$_3$**, with **1a** did not produce catalytically active species.

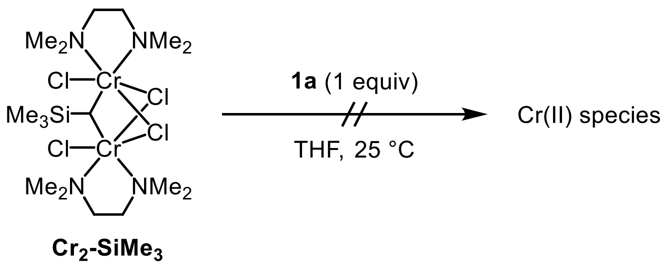

(S2)

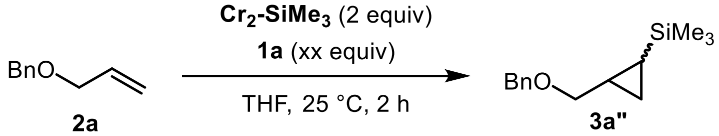

(S3)

xx = 0: 49% yield, *trans* : *cis* = 63 : 37
xx = 2: 50% yield, *trans* : *cis* = 66 : 34

Reactivity of Organosilicon-based Reductant **1a**

We checked the reactivity of **1a** with bromoform (eq. S4); however, no reaction was observed by NMR analysis in THF-d_8. The direct activation of bromoform by **1a** was excluded from a plausible reaction pathway for generating carbene species.

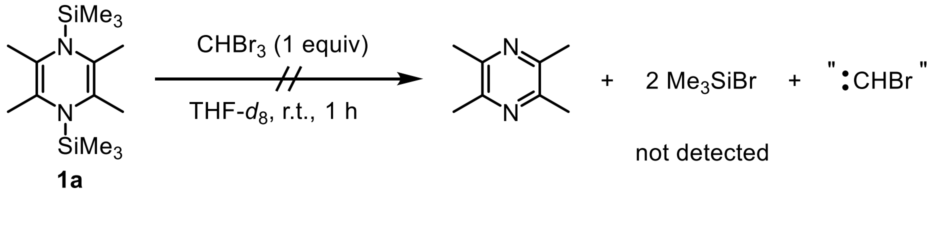

(S4)

In addition, the yield of **3a** was not improved when a mixture of $CHBr_3$ and **1a** was pre-treated by stirring at 50 °C for 1 hour before adding the chromium catalyst (Scheme S1a and 1b), also indicating that the reaction of **1a** with $CHBr_3$ was not included in the catalytic cycle.

Scheme S1. Effect of Pre-treatment of $CHBr_3$ and **1a**

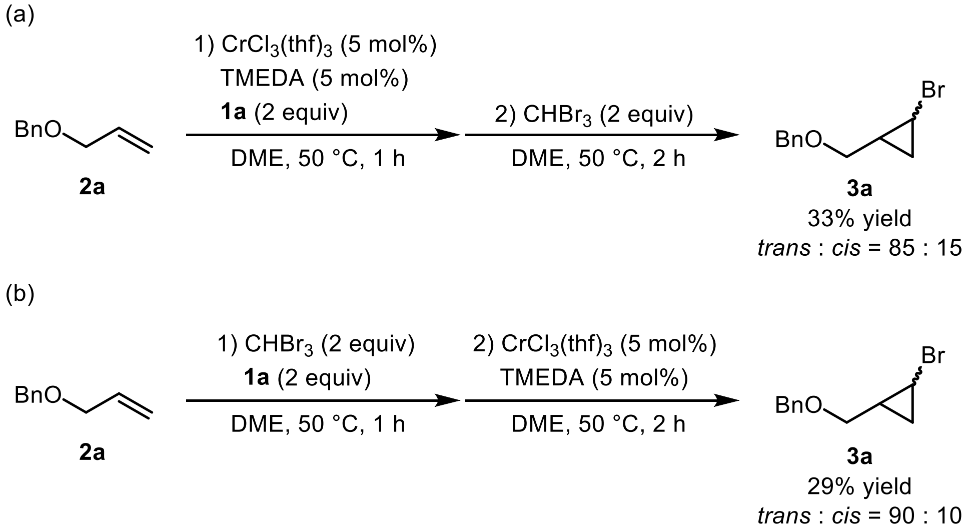

7. X-ray Diffraction Analysis

The crystal of *trans*-**3c** were mounted on the CryoLoop (Hampton Research Corp) with a layer of light mineral oil and placed in a nitrogen stream at 113(1) K. All measurements were made on a Rigaku XtaLAB P200 diffractometer using multi-layer mirror monochromated Mo-Kα (0.71075 Å) radiation. The structures were solved by SHELXS-2013,[7] and refined on F^2 by full-matrix least-squares method, using SHELXL-2013.[8] Non-hydrogen atoms were anisotropically refined. H-atoms were included in the refinement on calculated positions riding on their carrier atoms. The function minimized was $[\Sigma w(Fo^2 - Fc^2)^2]$ ($w = 1/[\sigma^2(Fo)^2 + (aP)^2 + bP]$), where $P = (\mathrm{Max}(Fo^2, 0) + 2Fc^2)/3$ with $\sigma^2(Fo^2)$ from counting statistics. The function $R1$ and $wR2$ were $(\Sigma||Fo| - |Fc||)/\Sigma|Fo|$ and $[\Sigma w(Fo^2 - Fc^2)^2/\Sigma(wFo^4)]^{1/2}$, respectively. The ORTEP-3 program[9] was used to draw the molecule.

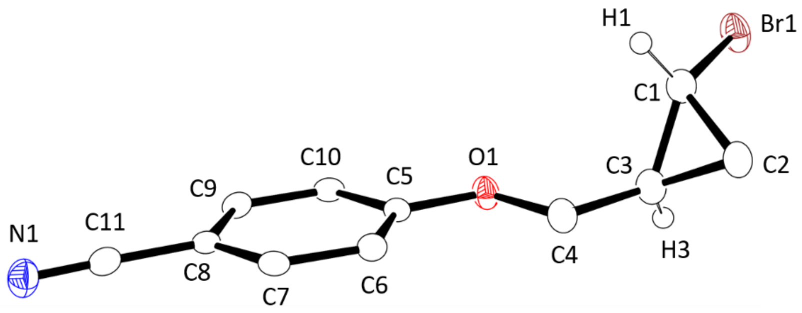

Figure S3. ORTEP drawing of *trans*-**3c** with 50% thermal ellipsoid. All the hydrogen atoms except for H1 and H3 are omitted for clarity. Selected bond lengths [Å] and bond angles [°]; C1—C2 : 1.487(4), C1—C3 : 1.497(4), C2—C3 : 1.507(3), Br1—C1 : 1.925(3), C3—C4 : 1.501(4), C4—O1 : 1.436(3), C5—O1 : 1.366(3), C11—N1 : 1.147(3)

Table S3. Crystal Data and Data Collection Parameters of *trans*-**3c**

	trans-**3c**
empirical formula	$C_{11}H_{10}BrNO$
formula weight	252.11
crystal system	triclinic
space group	$P\bar{1}$ (#2)
a, Å	6.7320(7)
b, Å	7.2280(6)
c, Å	12.0162(17)
α, deg.	80.831(11)
β, deg.	86.517(11)
γ, deg.	62.902(8)
V, $Å^3$	513.81(11)
Z	2
D_{calcd}, g/cm^3	1.629
μ [Mo-*K*α], mm^{-1}	3.9761
T, K	113(2)
crystal size, mm	0.480 x 0.400 x 0.250
θ range for data collection (deg.)	3.153 to 27.525
no. of reflections measured	8391
unique data (*R*int)	2353 (0.0588)
data / restraints / parameters	2353 / 0 / 127
*R*1 (I > 2.0σ(I))	0.0286
*wR*2 (I > 2.0σ(I))	0.0690
*R*1 (all data)	0.0327
*wR*2 (all data)	0.0690
GOF on F^2	0.988
Δρ, e $Å^{-3}$	0.92, -0.63

a) $R1 = (\Sigma||Fo| - |Fc||)/\Sigma|Fo|$, b) $wR2 = [\Sigma w(Fo^2 - Fc^2)^2/\Sigma(wFo^4)]^{1/2}$

8. References

(1) (a) M. Murai, R. Taniguchi, N. Hosokawa, Y. Nishida, H. Mimachi, T. Oshiki, K. Takai, *J. Am. Chem. Soc.,* 2017, **139**, 13184; (b) D. Werner, R. Anwander, *J. Am. Chem. Soc.,* 2018, **140**, 14334.

(2) A. P. Cole, V. Mahadevan, L. M. Mirica, X. Ottenwaelder, T. D. P. Stack, *Inorg. Chem.,* 2005, **44**, 7345.

(3) K. Takai, Y. Kunisada, Y. Tachibana, N. Yamaji, E. Nakatani, *Bull. Chem. Soc. Jpn.,* 2004, **77**, 1581.

(4) T. Saito, H. Nishiyama, H. Tanahashi, K. Kawakita, H. Tsurugi, K. Mashima, *J. Am. Chem. Soc.,* 2014, **136**, 5161.

(5) (a) B. Alcaide, P. Almendros, J. M. Alonso, M. F. Aly, *Org. Lett.,* 2001, **3**, 3781.; (b) J. Rudolph, L. Chen, D. Majumdar, W. H. Bullock, M. Burns, T. Claus, F. E. D. Cruz, M. Daly, F. J. Ehrgott, J. S. Johnson, J. N. Livingston, R. W. Schoenleber, J. Shapiro, L. Yang, M. Tsutsumi, X. Ma, *J. Med. Chem.,* 2007, **50**, 984.; (c) W. Chen, X.-D. Yang, Y. Li, L.-J. Yang, X.-Q. Wang, G.-L. Zhang, H.-B. Zhang, *Org. Biomol. Chem.,* 2011, **9**, 4250.; (d) X. Tan, L. Kong, H. Dai, X. Cheng, F. Liu, C. Tschierske, *Chem. Eur. J.,* 2013, **19**, 16303.; (e) C. Chardin, J. Rouden, S. Livi, J. Baudoux, *Green Chem.,* 2017, **19**, 5054.

(6) (a) J. Burés, *Angew. Chem., Int. Ed.,* 2016, **55**, 2028. (b) J. Burés, *Angew. Chem., Int. Ed.,* 2016, **55**, 16084.

(7) (a) G. M. Sheldrick, A Short History of SHELX, *Acta Crystallogr., Sect. A: Fundam. Crystallogr*., 2008, **64**, 112.; (b) A. Altomare, G. Cascarano, C. Giacovazzo, A. Guagliardi, M. C. Burla, G. Polidori, M. Camalli, SIR92 - A Program for Automatic Solution of Crystal Structures by Direct Methods, *J. Appl. Crystallogr*., 1994, **27**, 435.

(8) G. M. Sheldrick, *Acta Crystallogr., Sect. A: Fundam. Crystallogr*., 2008, **64**, 112.

(9) L. J. Farrugia, WinGX Suite for Small-Molecule SingleCrystal Crystallography, *J. Appl. Crystallogr*., 1999, **32**, 837.

9. NMR Spectra

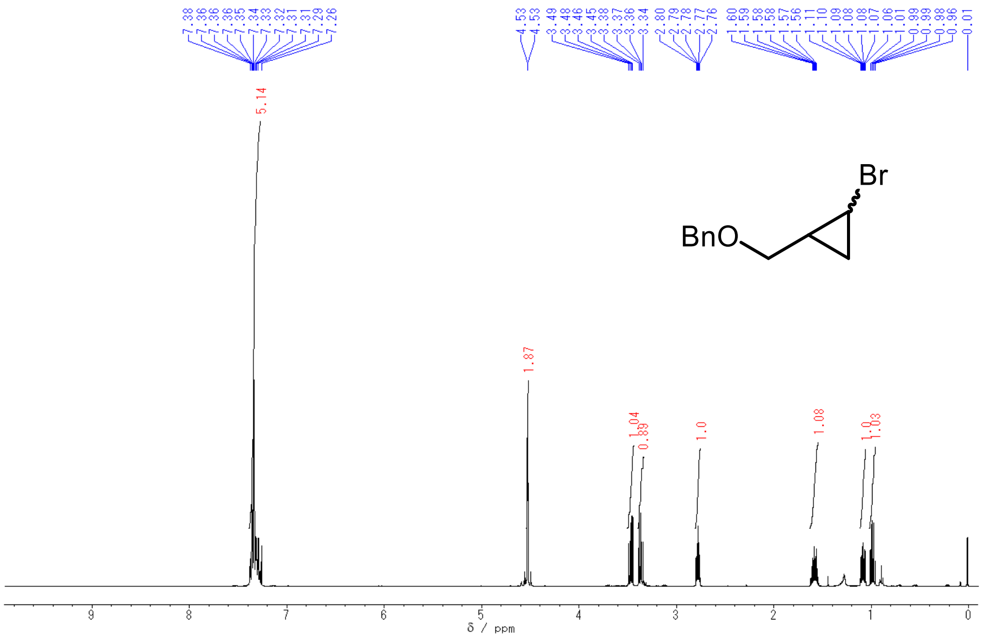

Figure S4. ^{1}H NMR spectrum of *trans*-**3a** in $CDCl_3$

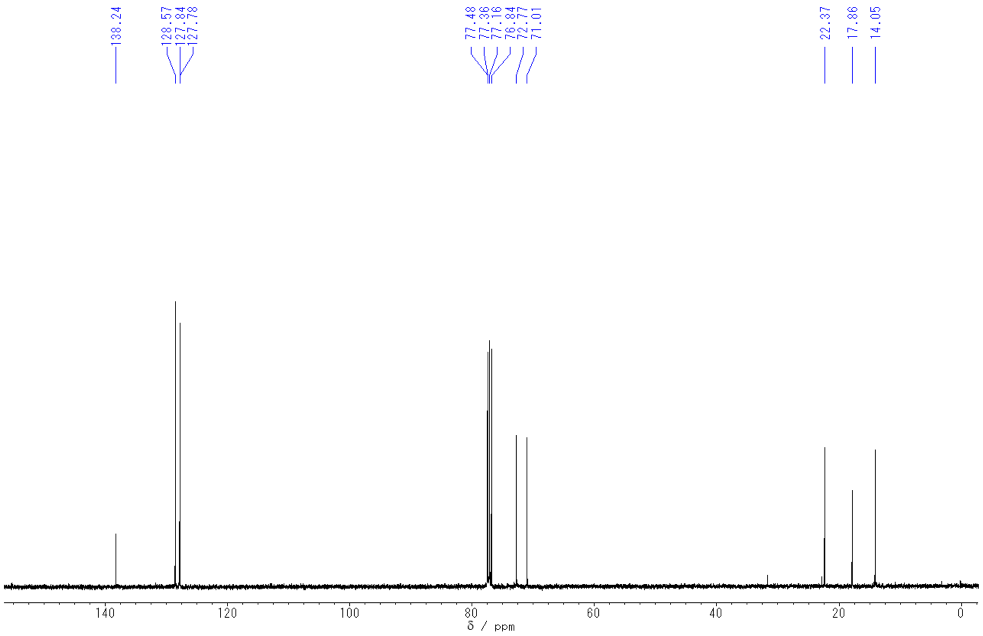

Figure S5. $^{13}C\{^{1}H\}$ NMR spectrum of *trans*-**3a** in $CDCl_3$

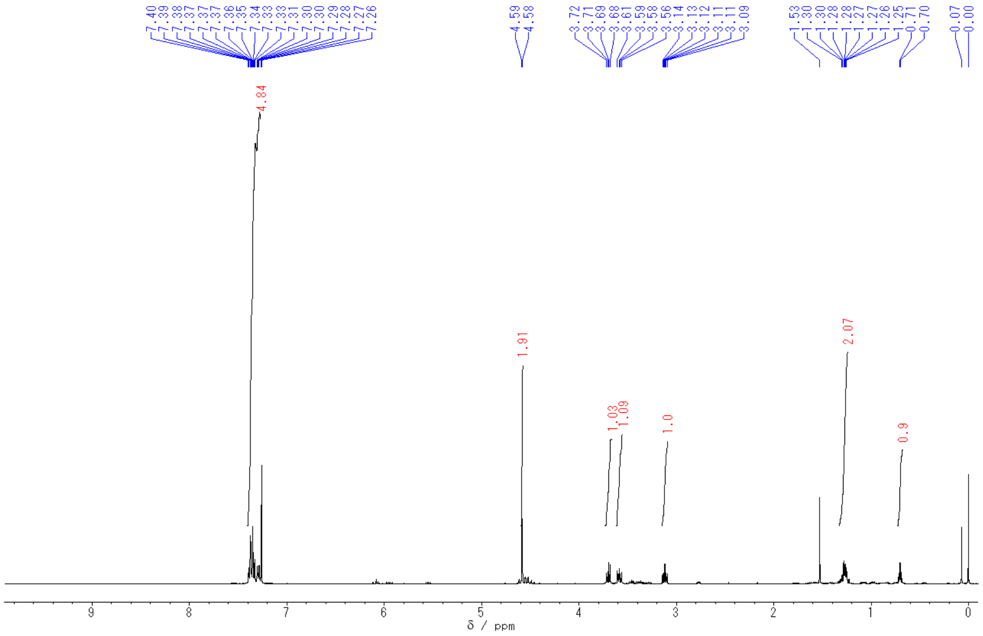

Figure S6. ^{1}H NMR spectrum of *cis*-**3a** in $CDCl_3$

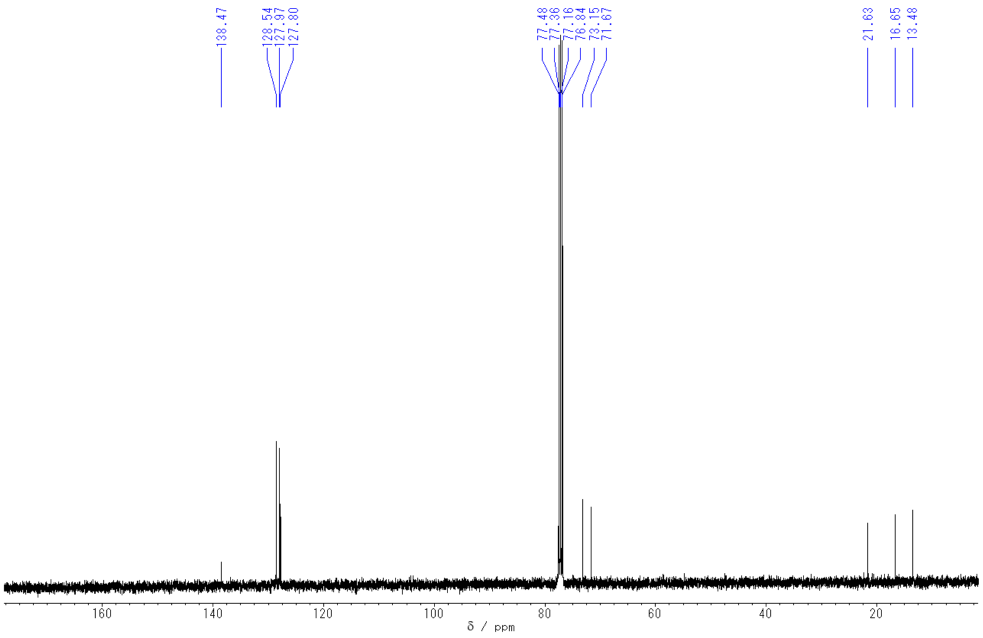

Figure S7. $^{13}C\{^{1}H\}$ NMR spectrum of *cis*-**3a** in $CDCl_3$

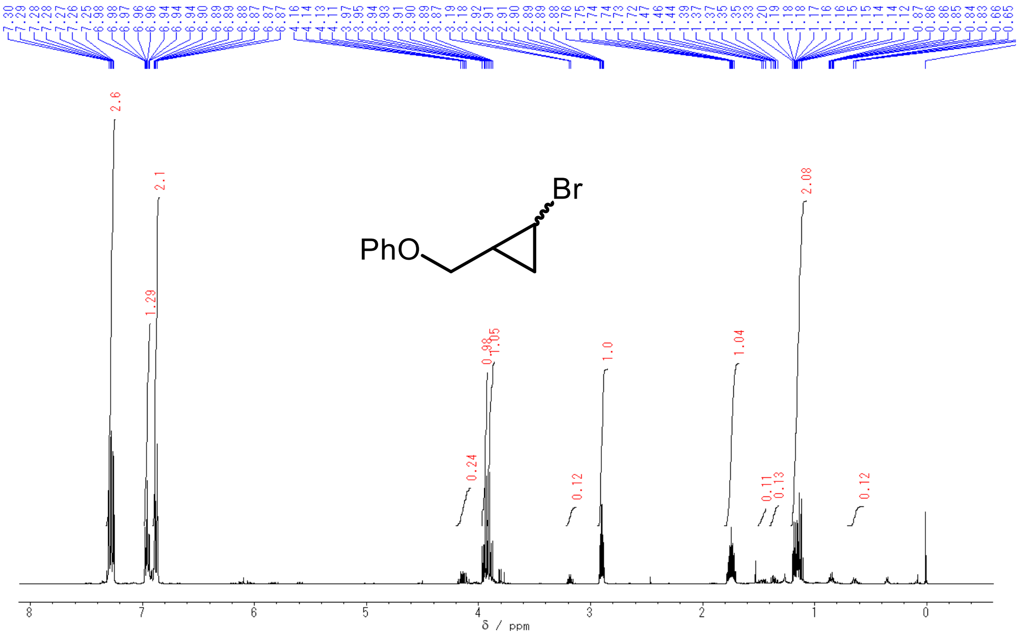

Figure S8. ^{1}H NMR spectrum of **3b** (*trans*/*cis* mixture) in $CDCl_3$

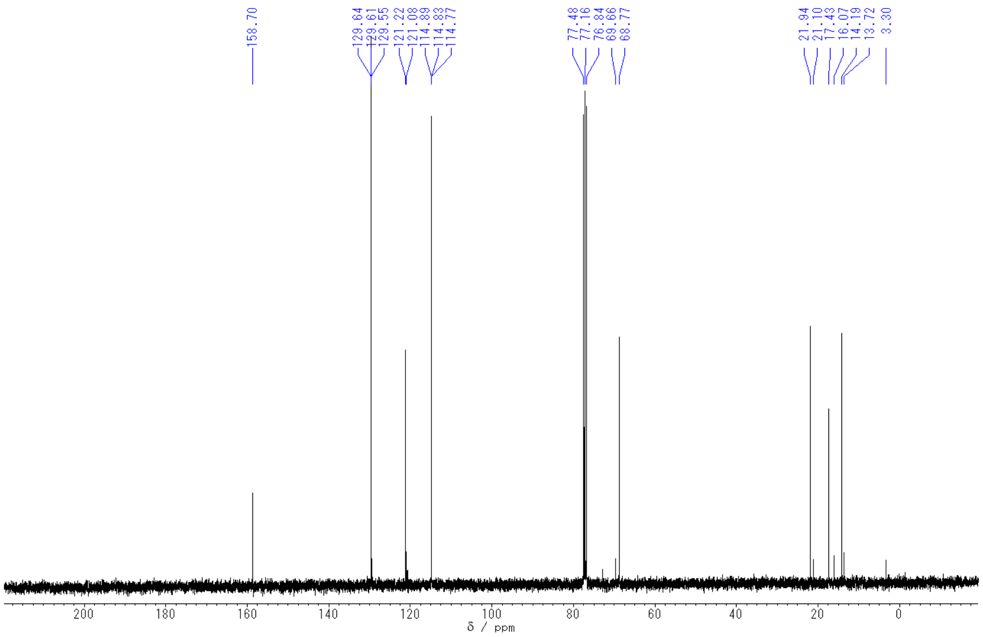

Figure S9. $^{13}C\{^{1}H\}$ NMR spectrum of **3b** (*trans*/*cis* mixture) in $CDCl_3$

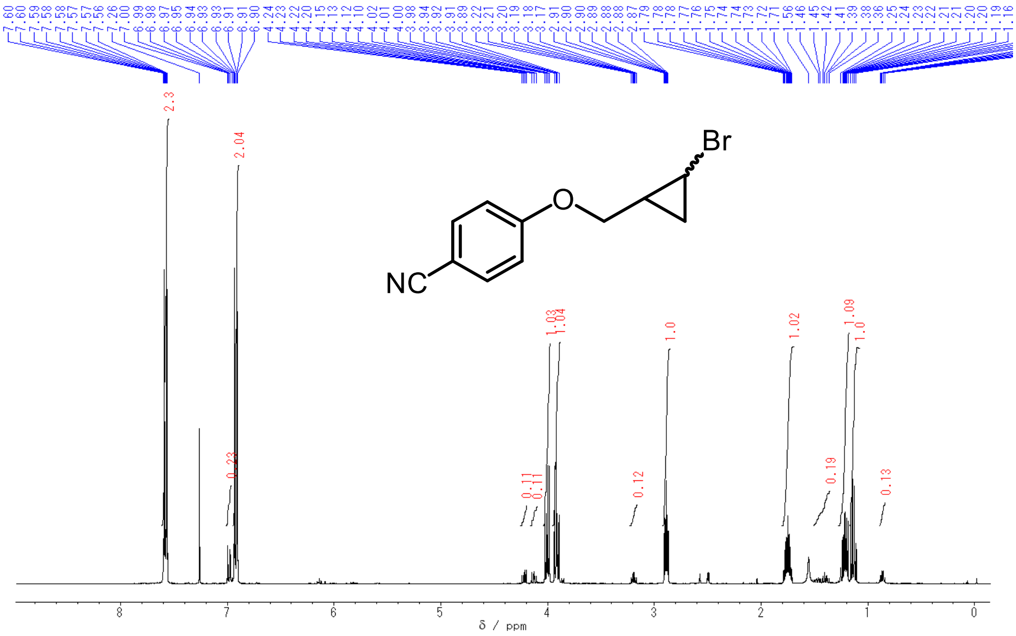

Figure S10. ^{1}H NMR spectrum of **3c** (*trans*/*cis* mixture) in $CDCl_3$

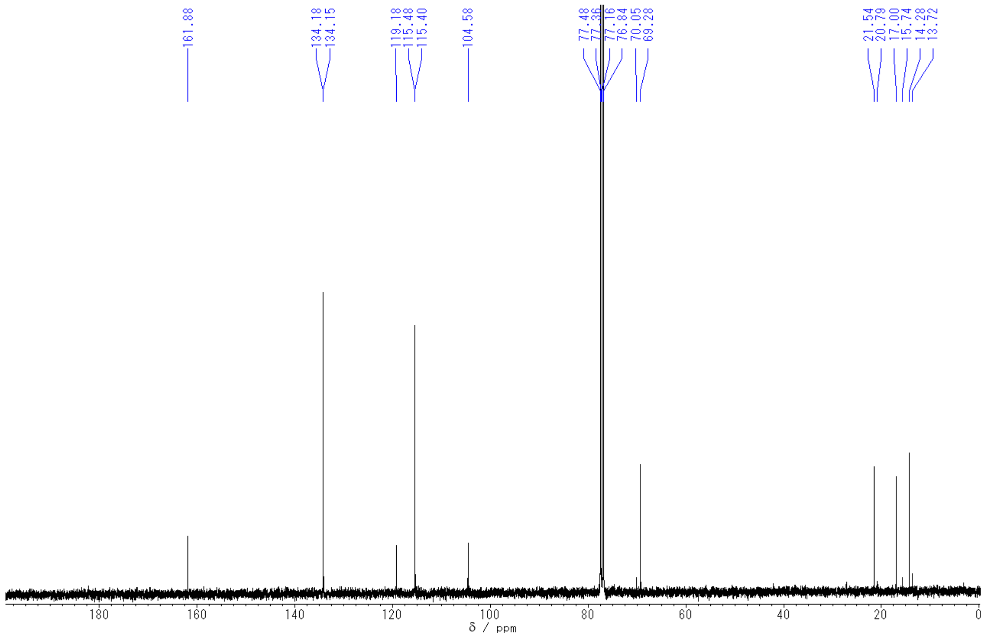

Figure S11. $^{13}C\{^{1}H\}$ NMR spectrum of **3c** (*trans*/*cis* mixture) in $CDCl_3$

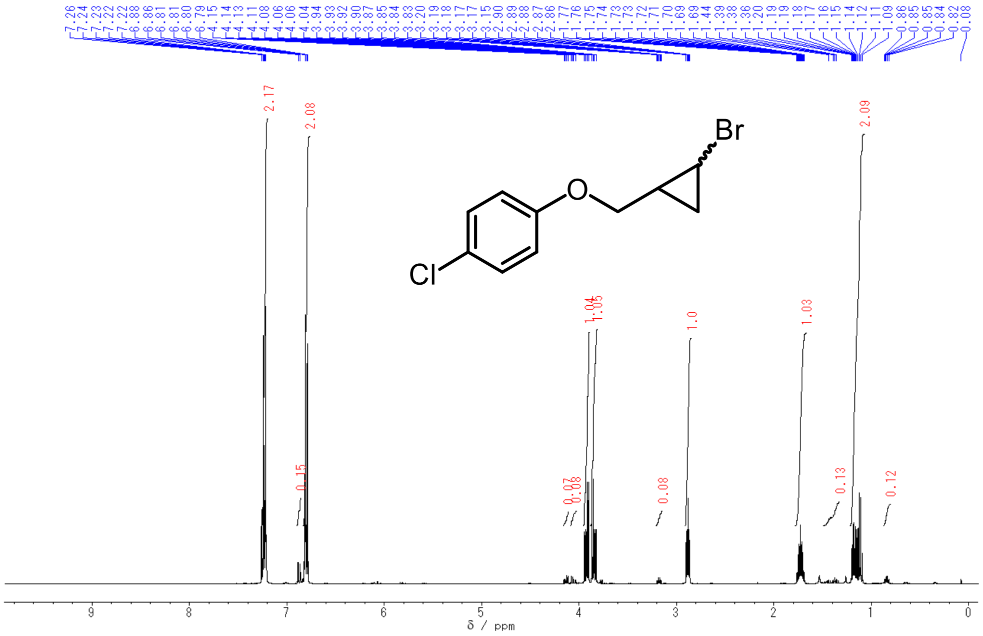

Figure S12. ^{1}H NMR spectrum of **3d** (*trans*/*cis* mixture) in $CDCl_3$

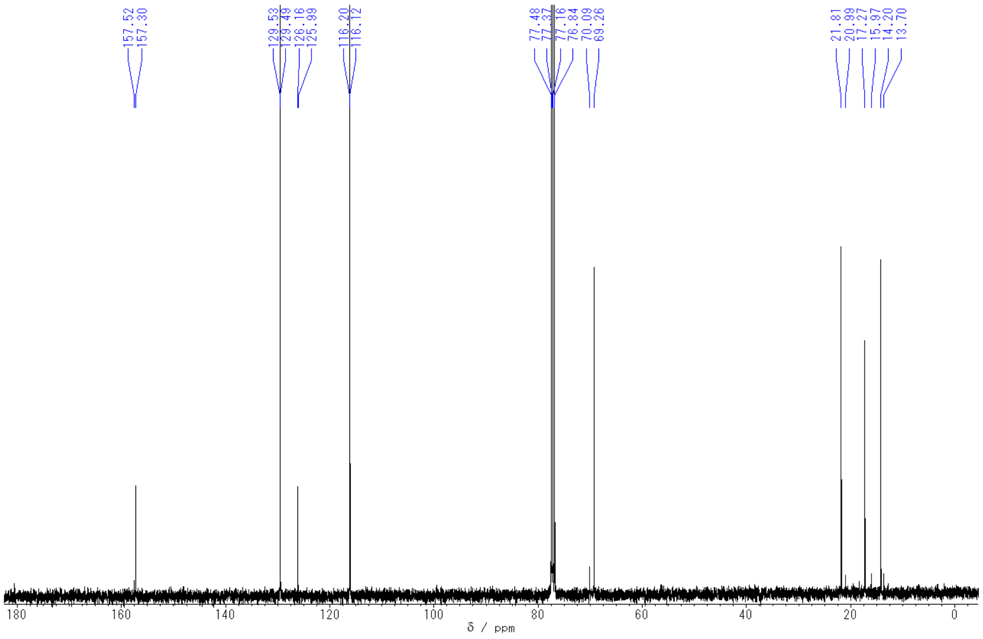

Figure S13. $^{13}C\{^{1}H\}$ NMR spectrum of **3d** (*trans*/*cis* mixture) in $CDCl_3$

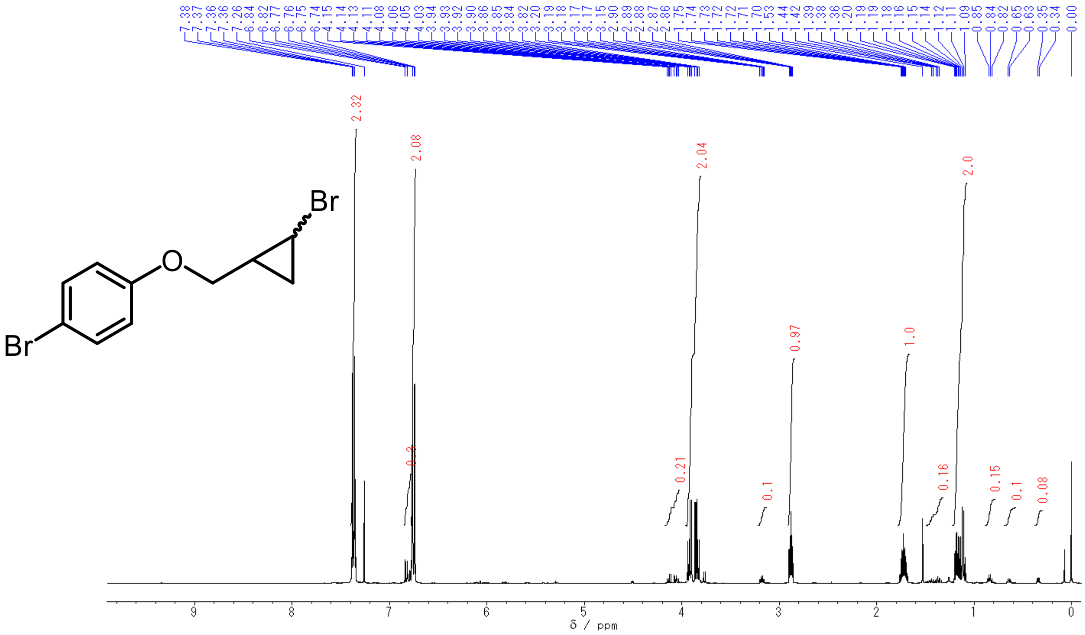

Figure S14. ^{1}H NMR spectrum of **3e** (*trans*/*cis* mixture) in $CDCl_3$

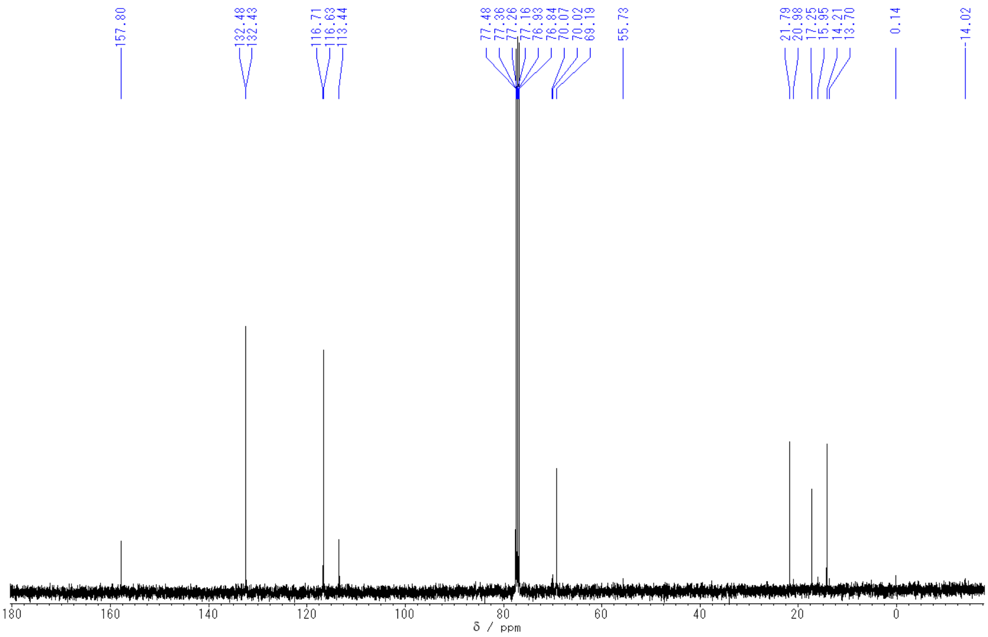

Figure S15. $^{13}C\{^{1}H\}$ NMR spectrum of **3e** (*trans*/*cis* mixture) in $CDCl_3$

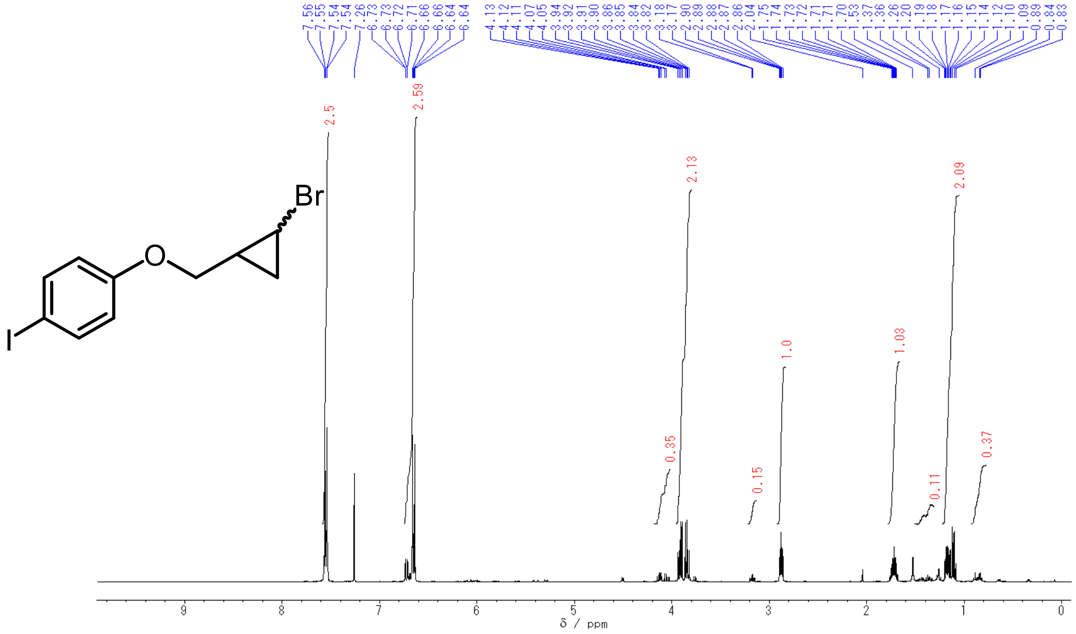

Figure S16. ^{1}H NMR spectrum of **3f** (*trans*/*cis* mixture) in $CDCl_3$

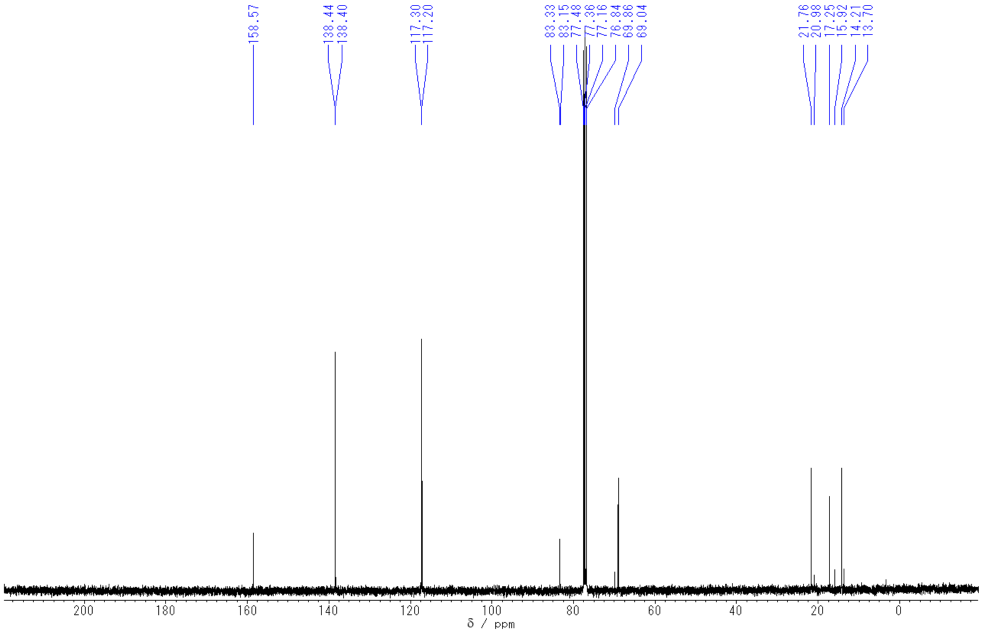

Figure S17. $^{13}C\{^{1}H\}$ NMR spectrum of **3f** (*trans*/*cis* mixture) in $CDCl_3$

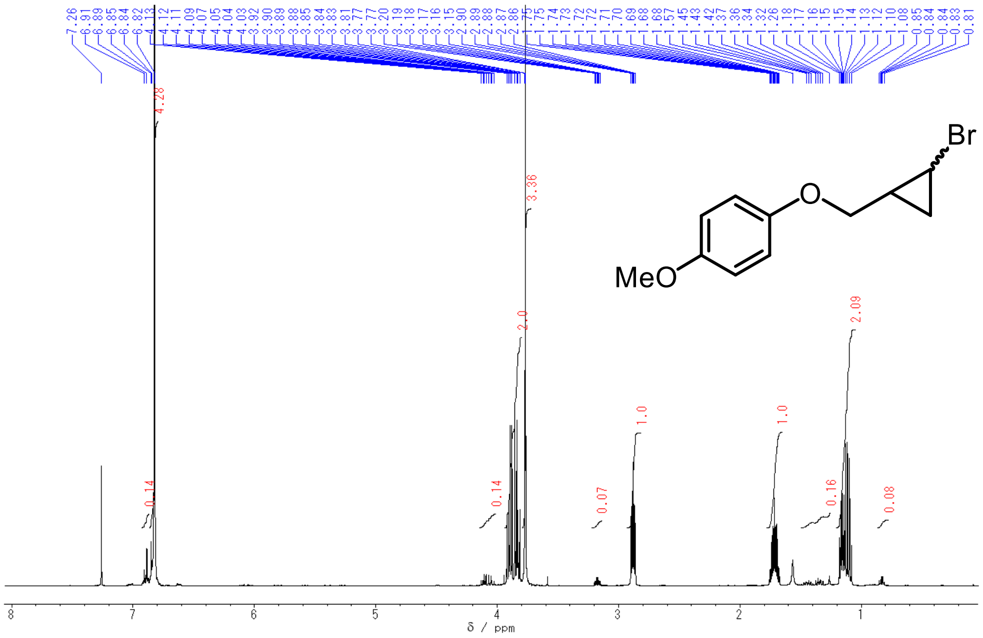

Figure S18. ^{1}H NMR spectrum of **3g** (*trans*/*cis* mixture) in $CDCl_3$

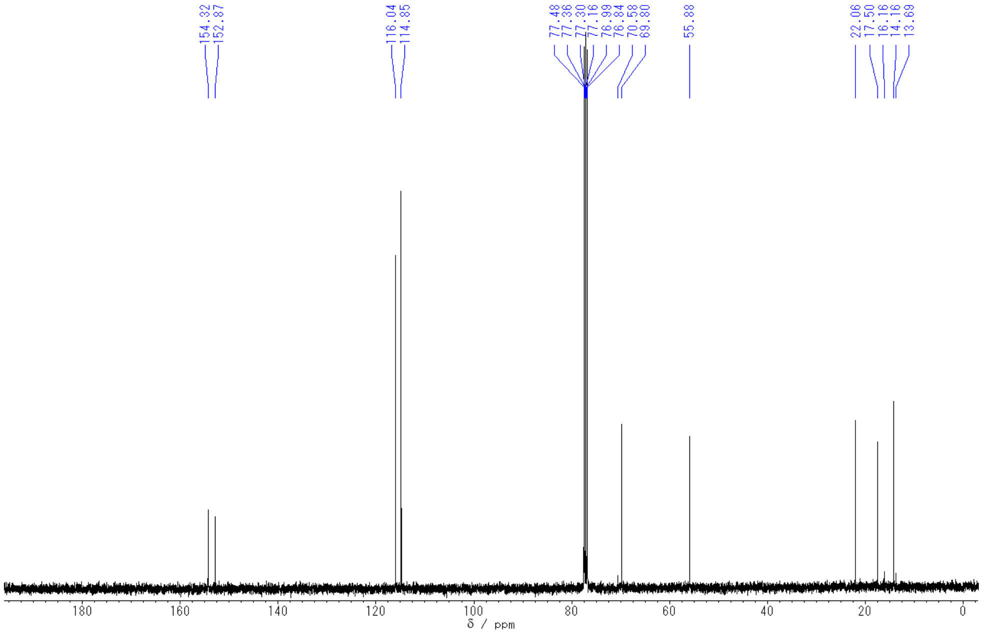

Figure S19. $^{13}C\{^{1}H\}$ NMR spectrum of **3g** (*trans*/*cis* mixture) in $CDCl_3$

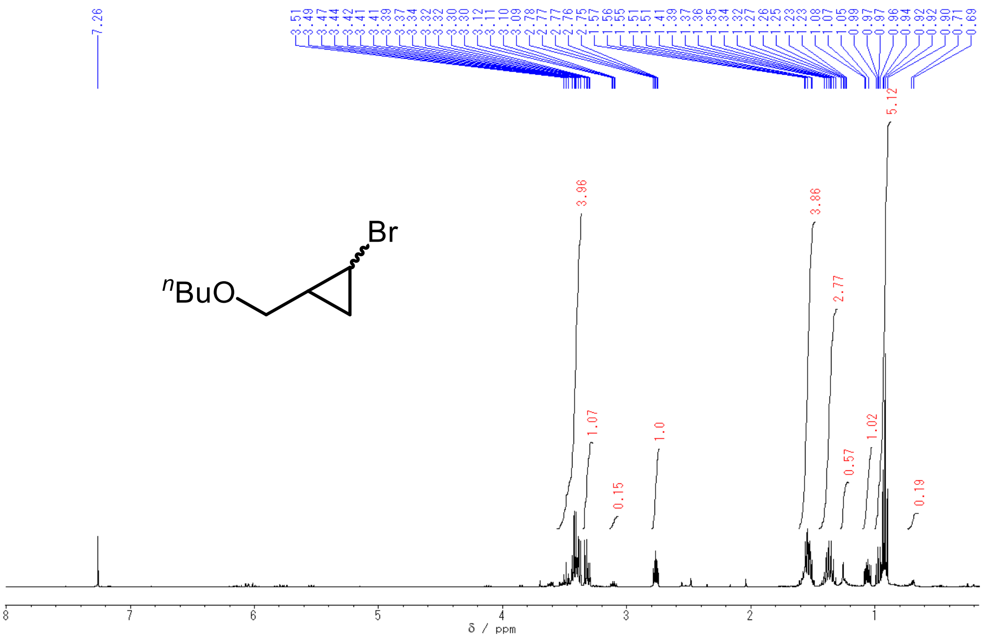

Figure S20. ^{1}H NMR spectrum of **3h** (*trans*/*cis* mixture) in $CDCl_3$

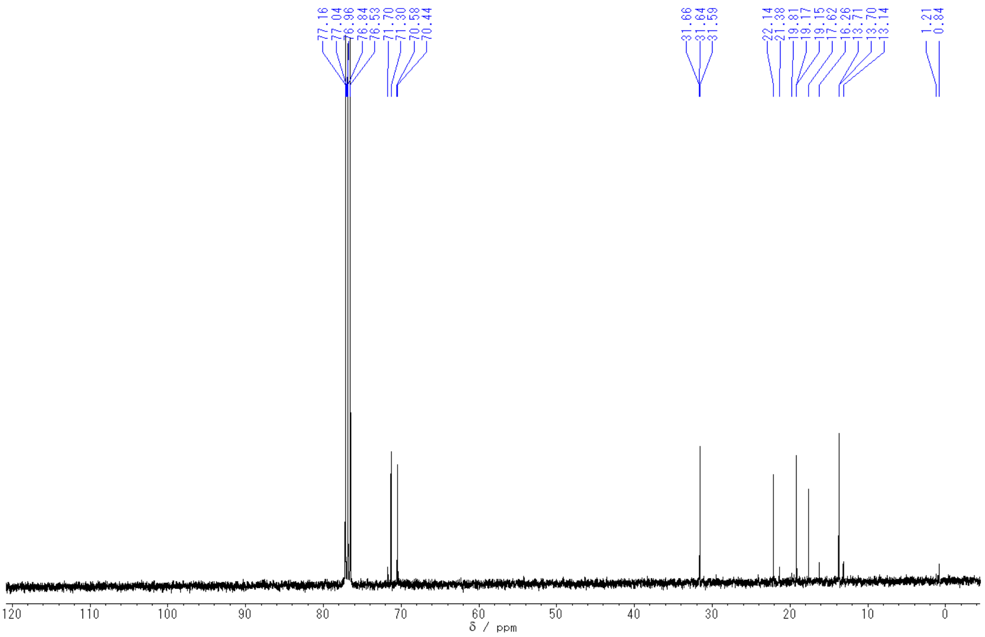

Figure S21. $^{13}C\{^{1}H\}$ NMR spectrum of **3h** (*trans*/*cis* mixture) in $CDCl_3$

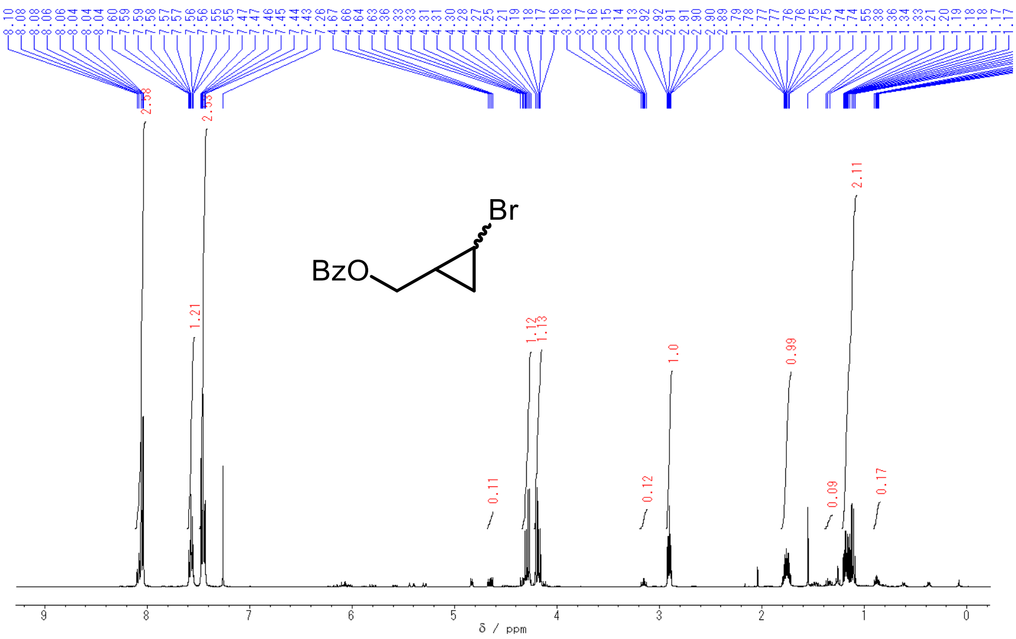

Figure S22. ^{1}H NMR spectrum of **3i** (*trans*/*cis* mixture) in $CDCl_3$

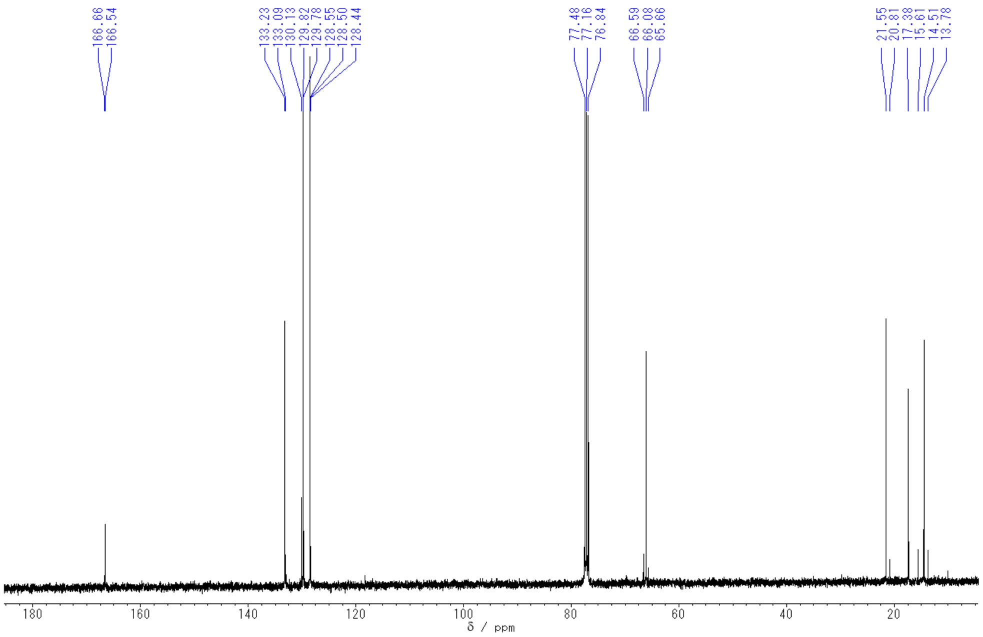

Figure S23. $^{13}C\{^{1}H\}$ NMR spectrum of **3i** (*trans*/*cis* mixture) in $CDCl_3$

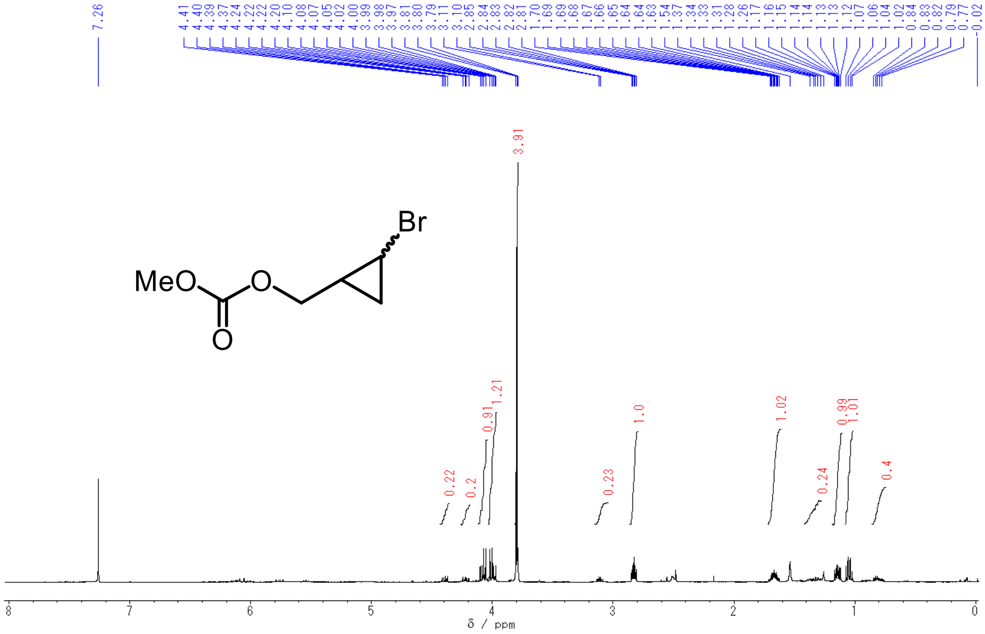

Figure S24. ^{1}H NMR spectrum of **3j** (*trans*/*cis* mixture) in $CDCl_3$

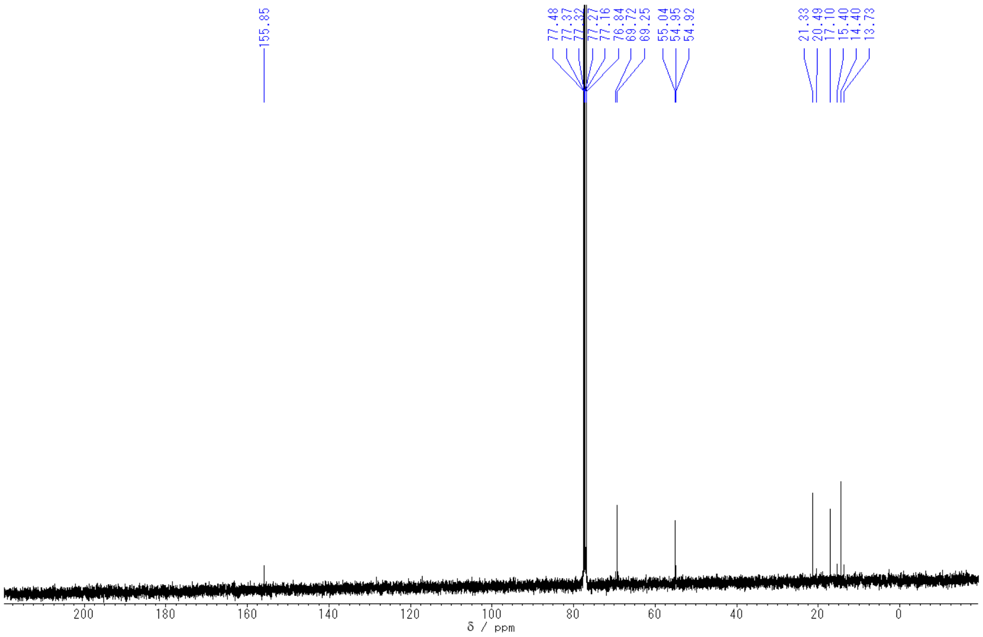

Figure S25. $^{13}C\{^{1}H\}$ NMR spectrum of **3j** (*trans*/*cis* mixture) in $CDCl_3$

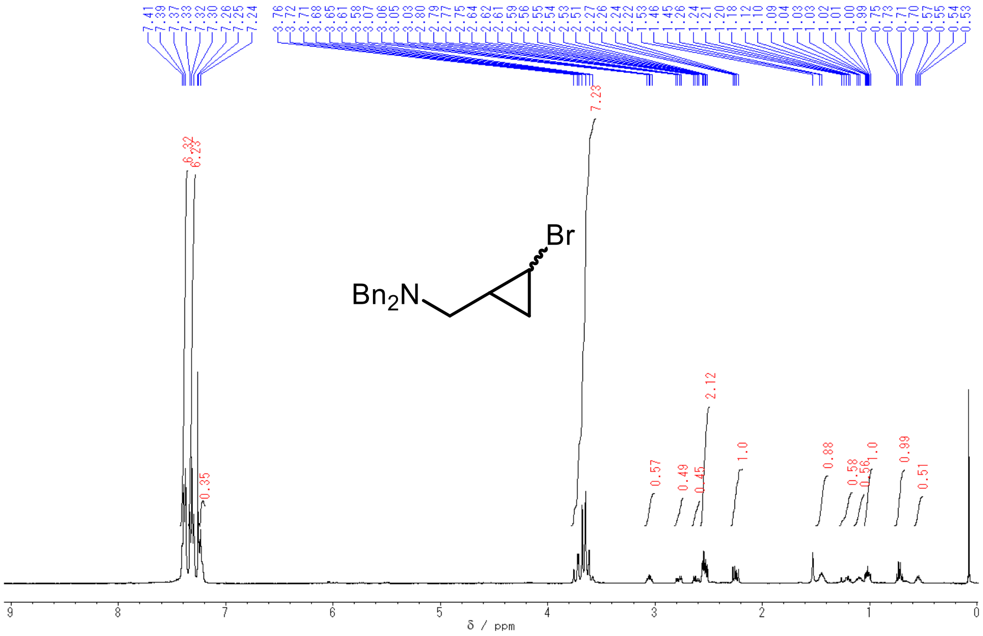

Figure S26. ^{1}H NMR spectrum of **3k** (*trans*/*cis* mixture) in $CDCl_3$

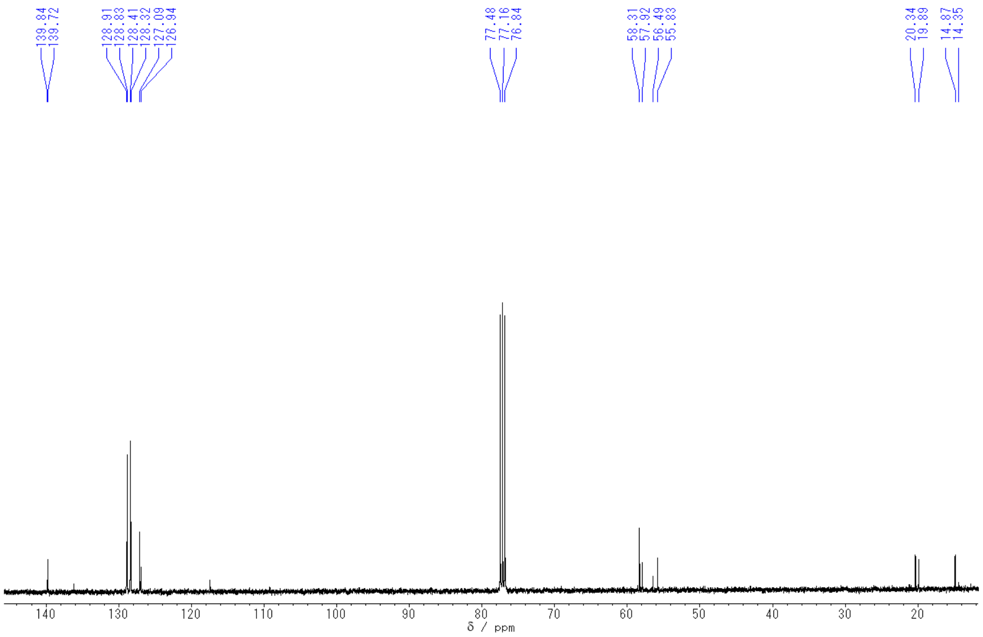

Figure S27. $^{13}C\{^{1}H\}$ NMR spectrum of **3k** (*trans*/*cis* mixture) in $CDCl_3$

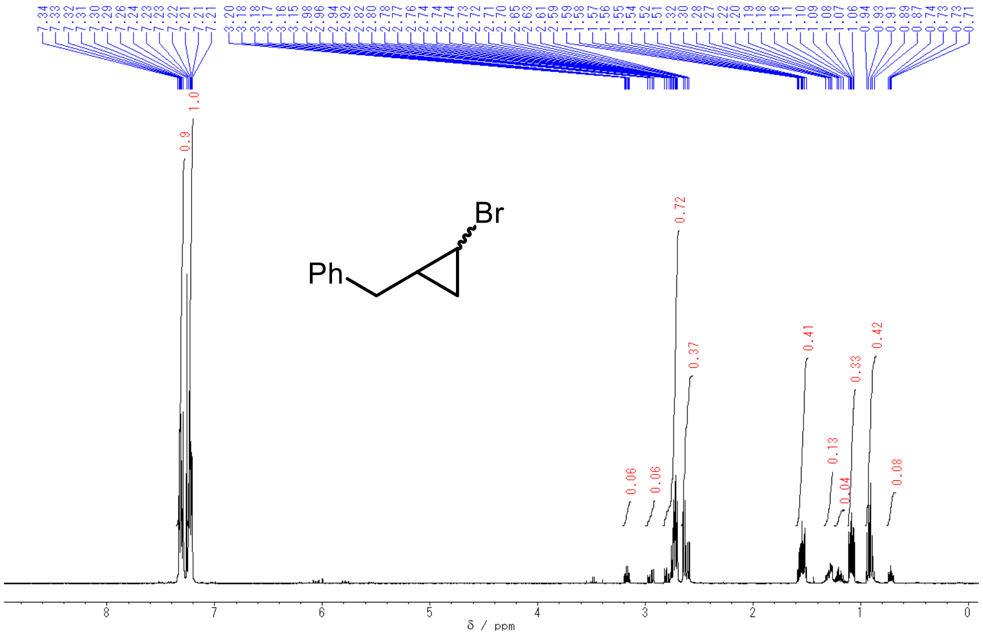

Figure S28. ^{1}H NMR spectrum of **3l** (*trans*/*cis* mixture) in $CDCl_3$

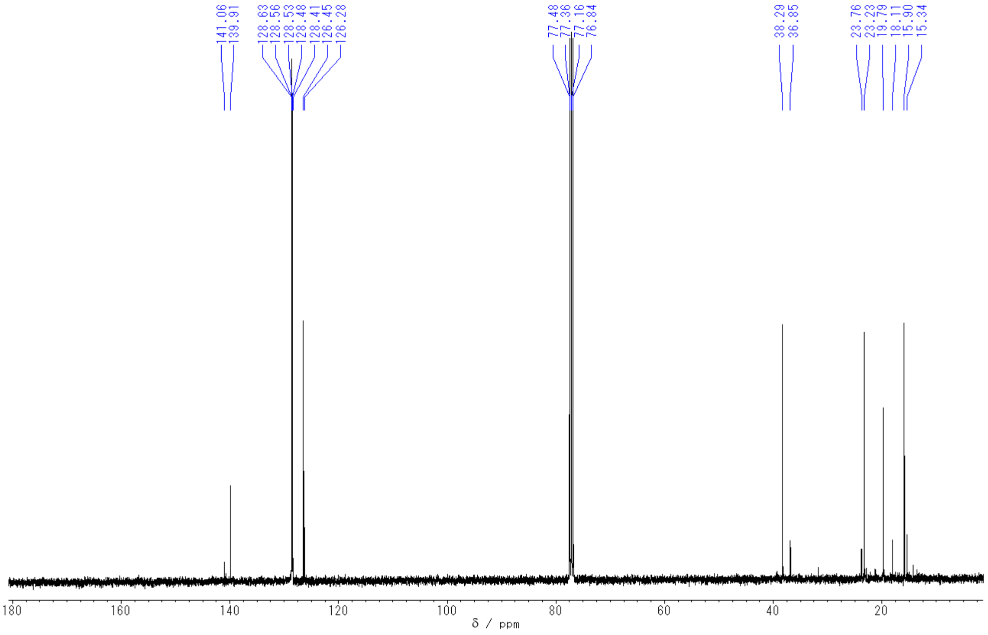

Figure S29. $^{13}C\{^{1}H\}$ NMR spectrum of **3l** (*trans*/*cis* mixture) in $CDCl_3$

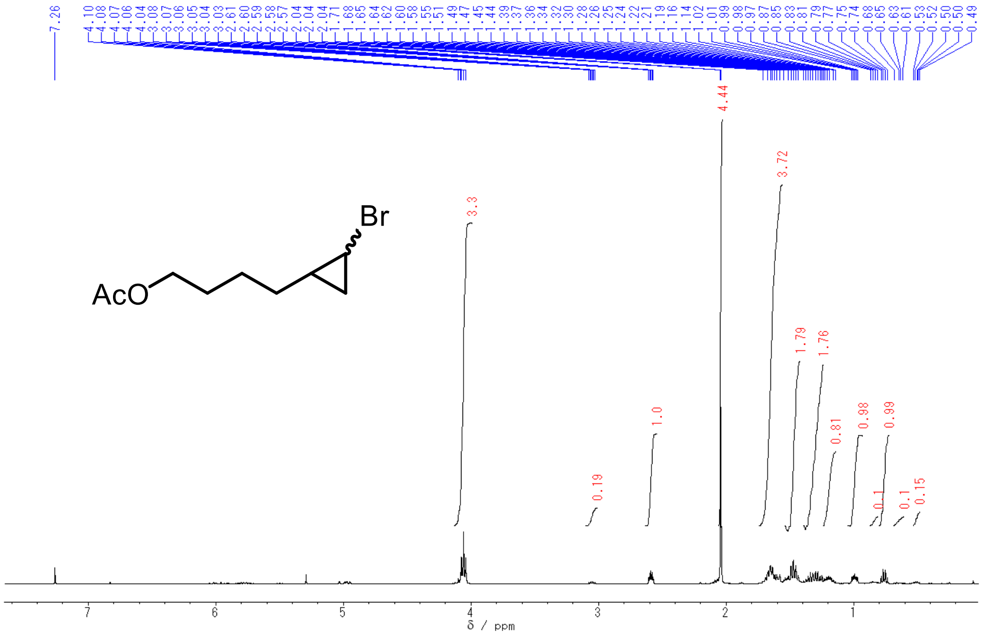

Figure S30. ^{1}H NMR spectrum of **3m** (*trans*/*cis* mixture) in $CDCl_3$

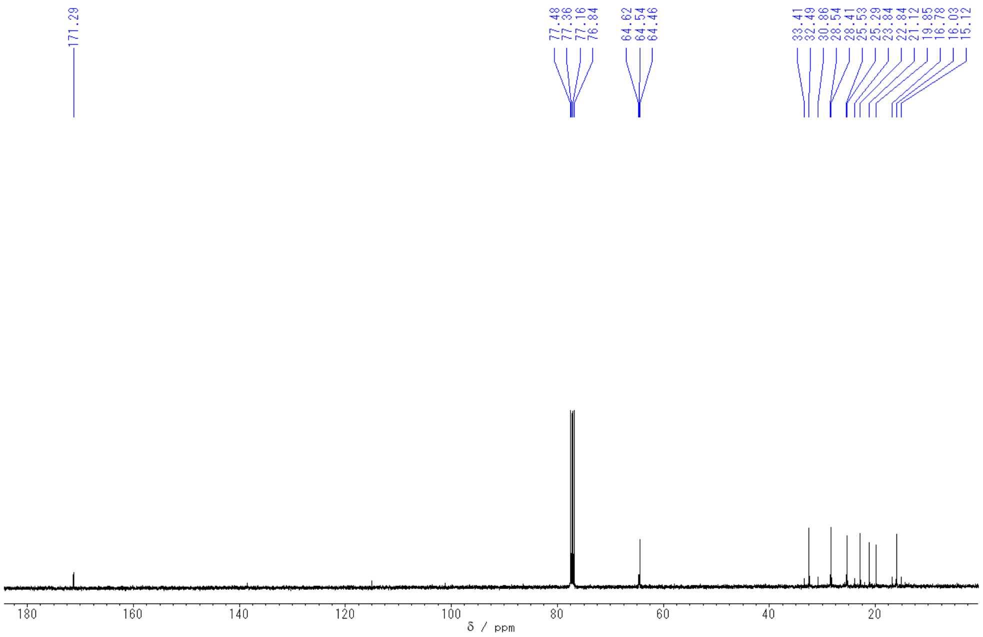

Figure S31. $^{13}C\{^{1}H\}$ NMR spectrum of **3m** (*trans*/*cis* mixture) in $CDCl_3$

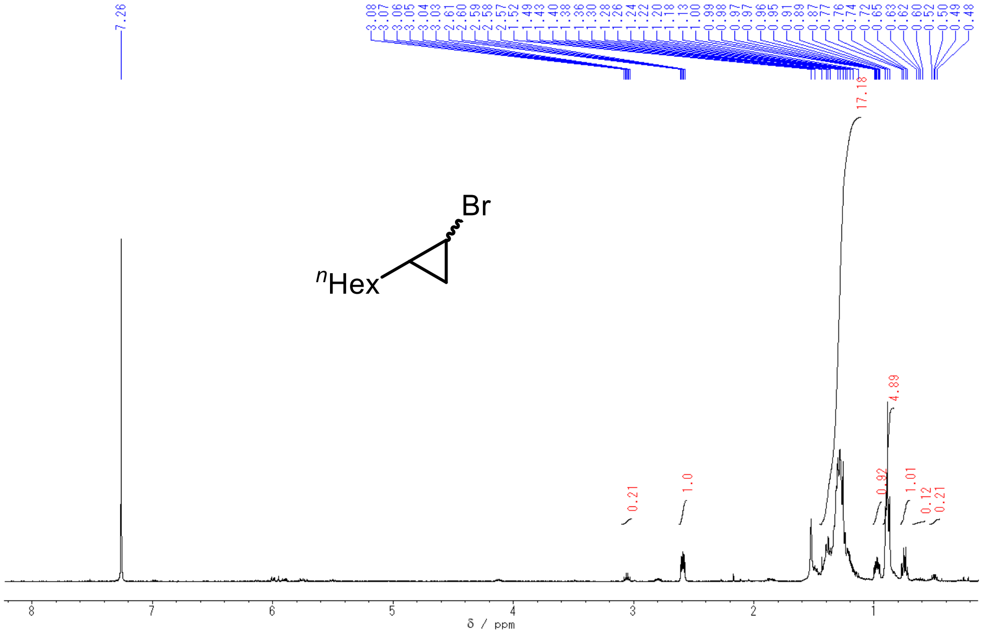

Figure S32. ^{1}H NMR spectrum of **3n** (*trans*/*cis* mixture) in $CDCl_3$

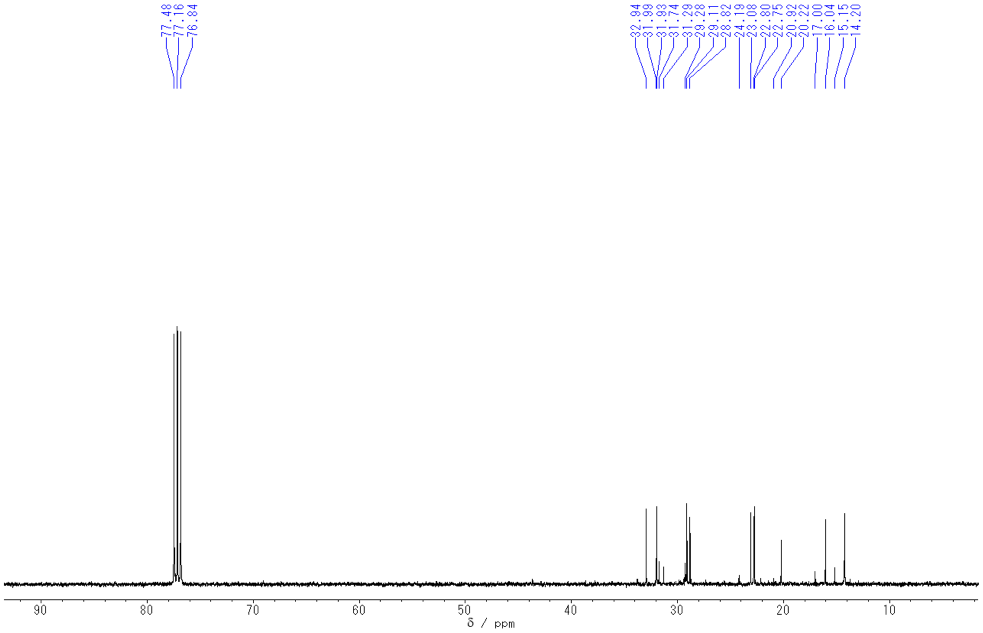

Figure S33. $^{13}C\{^{1}H\}$ NMR spectrum of **3n** (*trans*/*cis* mixture) in $CDCl_3$

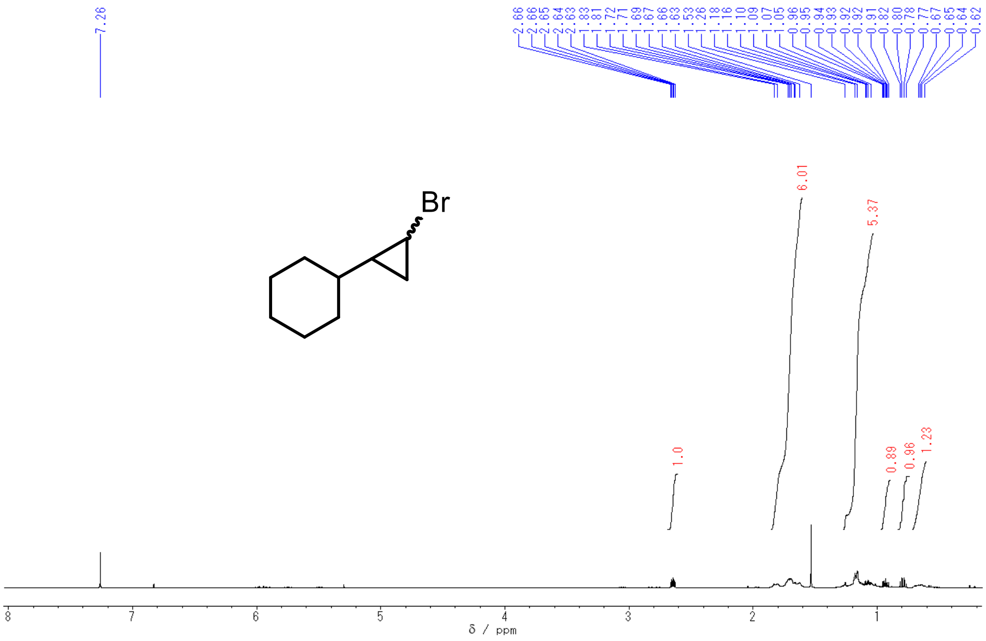

Figure S34. ^{1}H NMR spectrum of *trans*-**3o** in $CDCl_3$

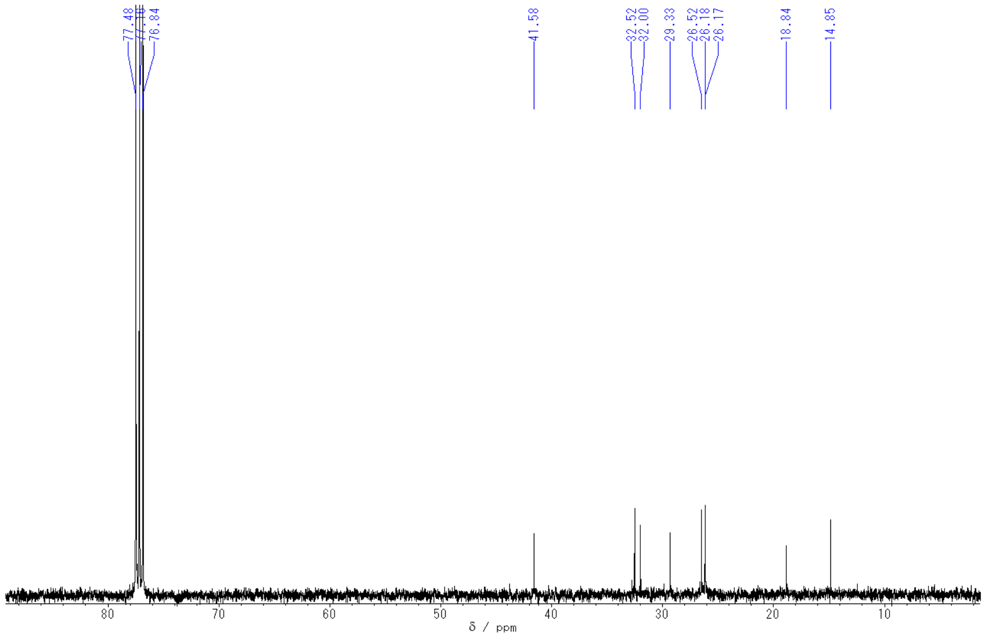

Figure S35. $^{13}C\{^{1}H\}$ NMR spectrum of *trans*-**3o** in $CDCl_3$

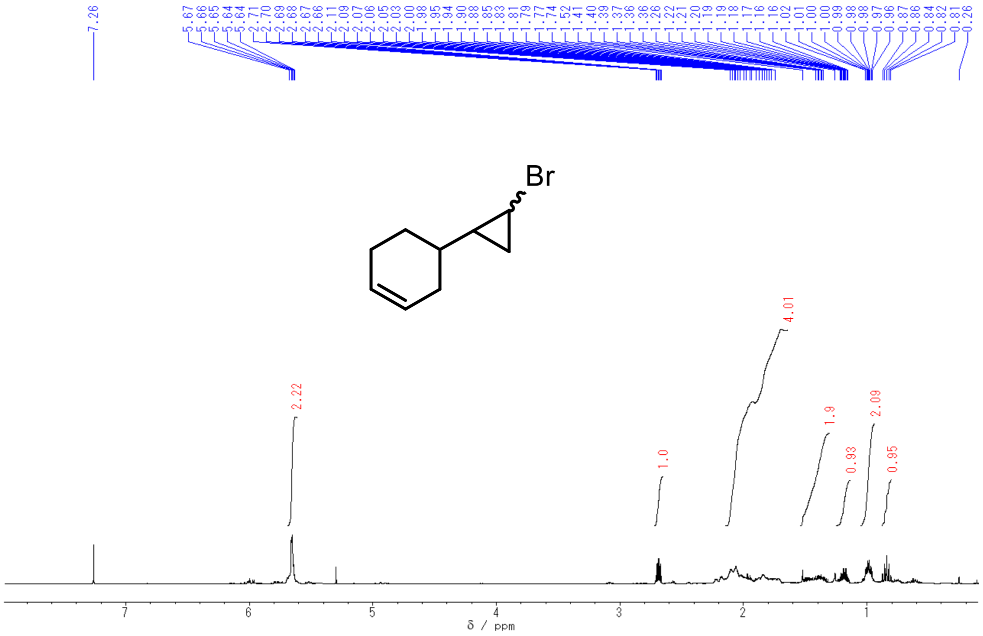

Figure S36. ^{1}H NMR spectrum of *trans*-**3p** in $CDCl_3$

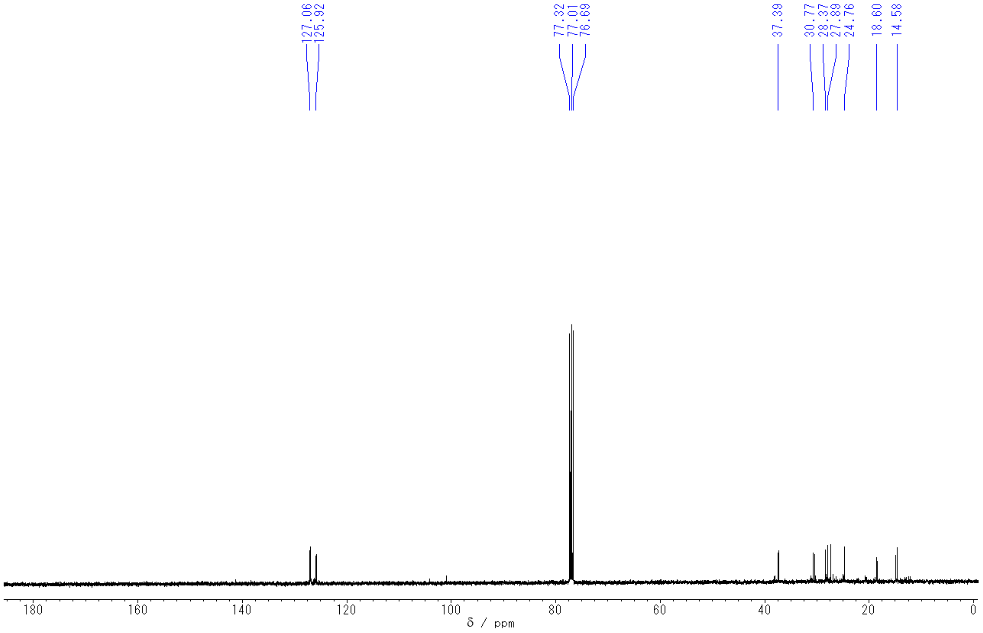

Figure S37. $^{13}C\{^{1}H\}$ NMR spectrum of *trans*-**3p** in $CDCl_3$

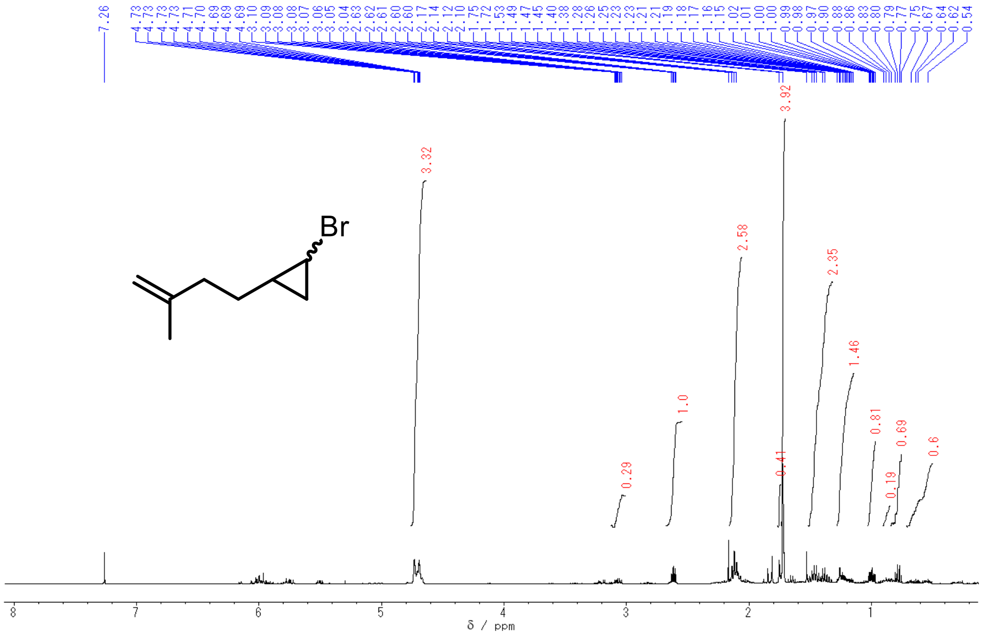

Figure S38. ^{1}H NMR spectrum of **3q** (*trans*/*cis* mixture) in $CDCl_3$

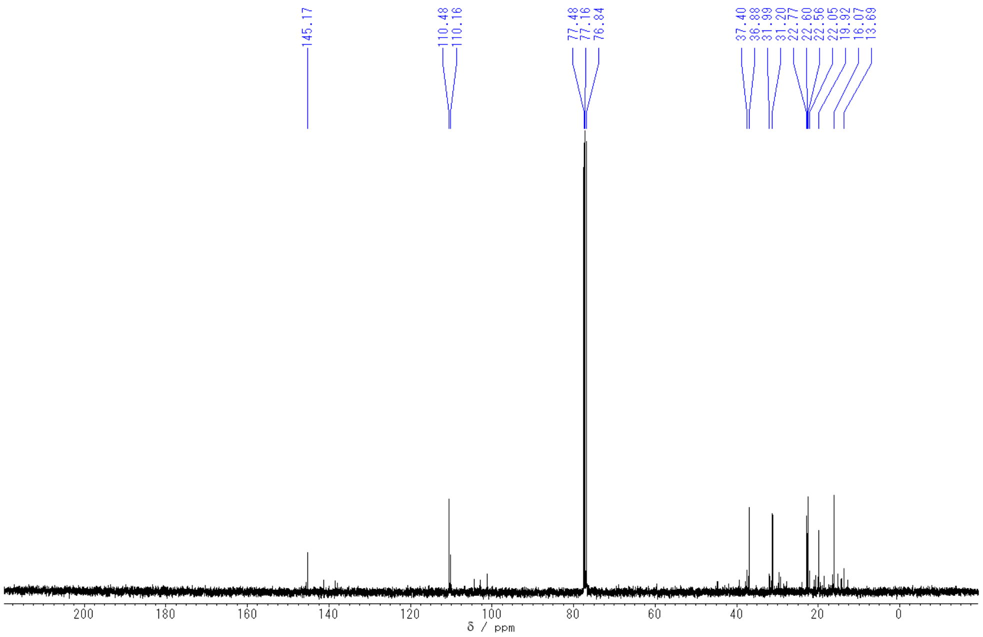

Figure S39. $^{13}C\{^{1}H\}$ NMR spectrum of **3q** (*trans*/*cis* mixture) in $CDCl_3$

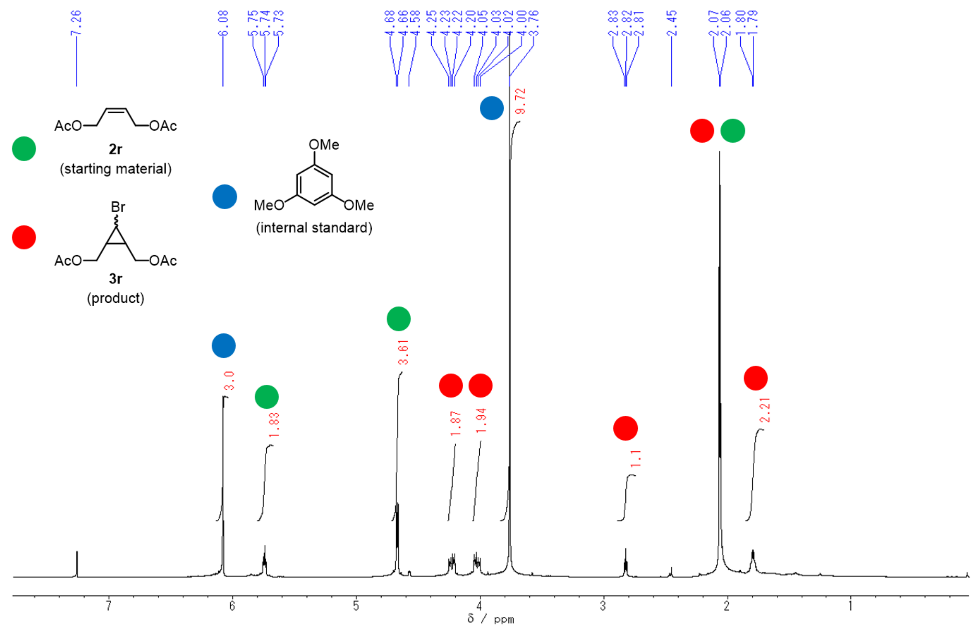

Figure S40. ^{1}H NMR spectrum of *trans*-**3r** in $CDCl_3$

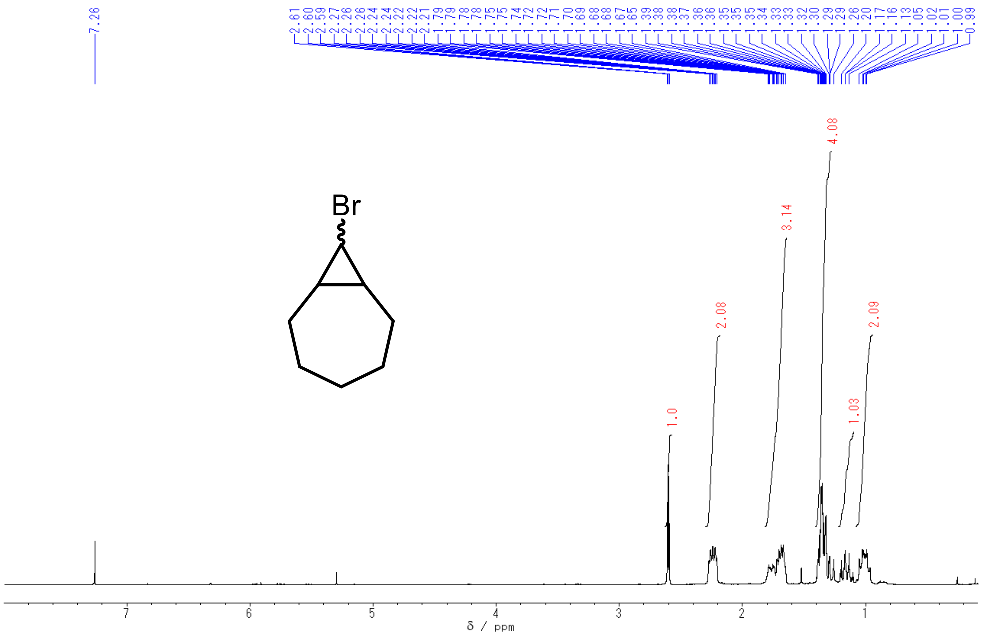

Figure S41. ^{1}H NMR spectrum of *trans*-**3s** in $CDCl_3$

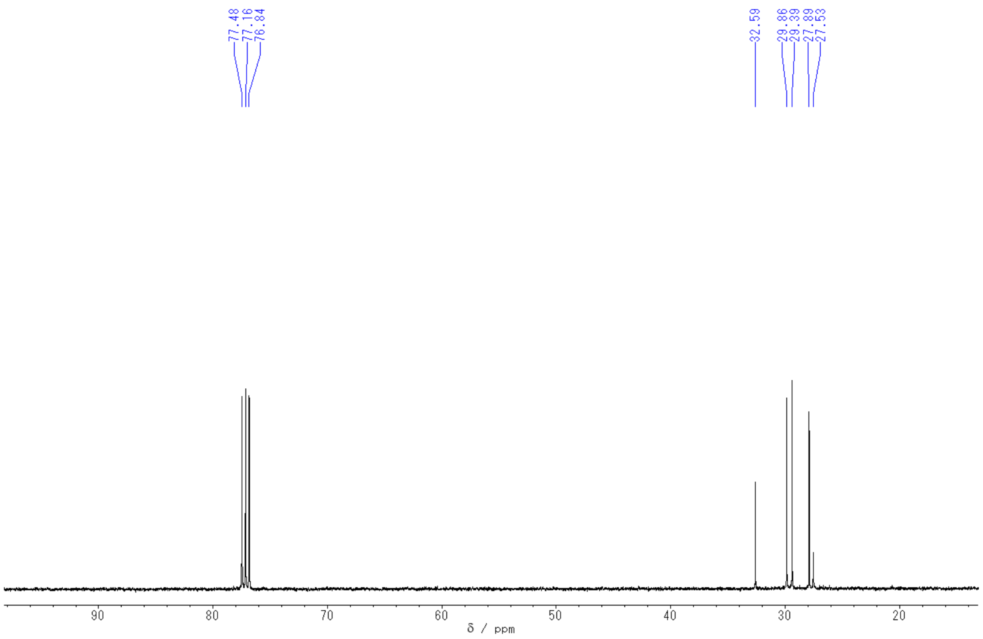

Figure S42. $^{13}C\{^{1}H\}$ NMR spectrum of *trans*-**3s** in $CDCl_3$

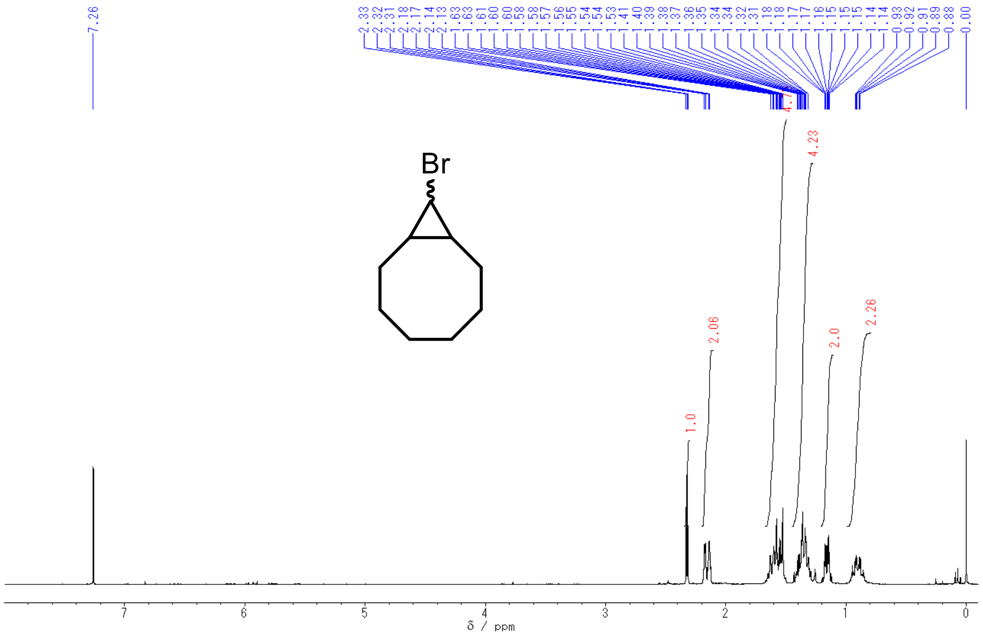

Figure S43. ^{1}H NMR spectrum of *trans*-**3t** in $CDCl_3$

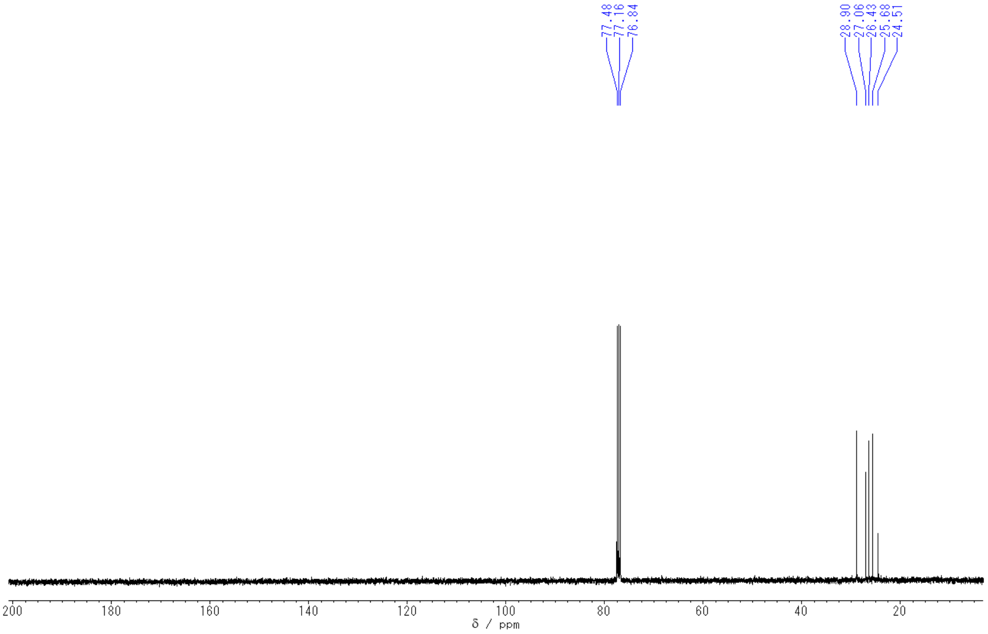

Figure S44. $^{13}C\{^{1}H\}$ NMR spectrum of *trans*-**3t** in $CDCl_3$

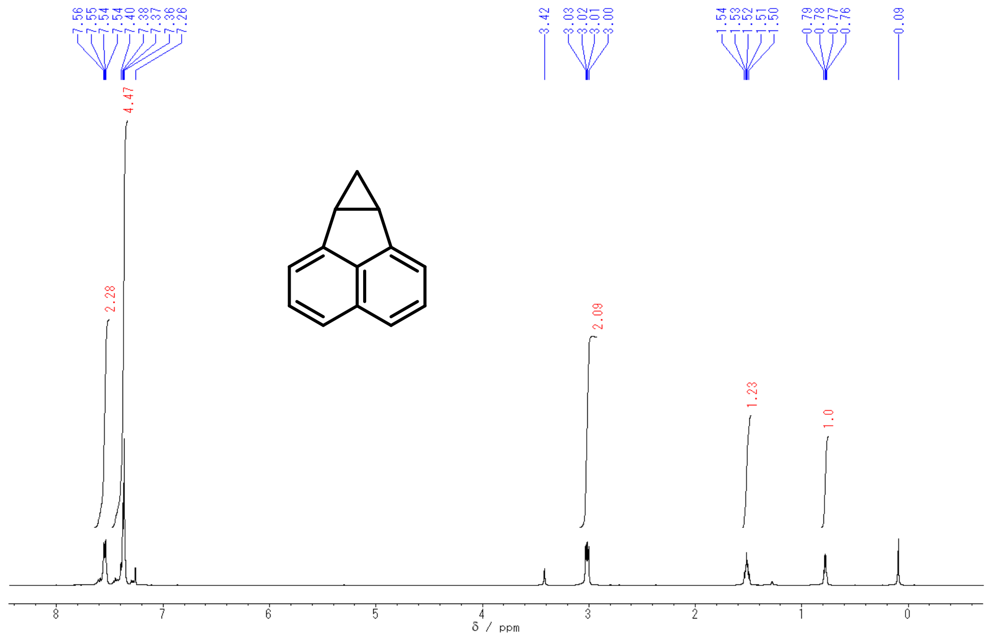

Figure S45. ^{1}H NMR spectrum of **3u** in $CDCl_3$

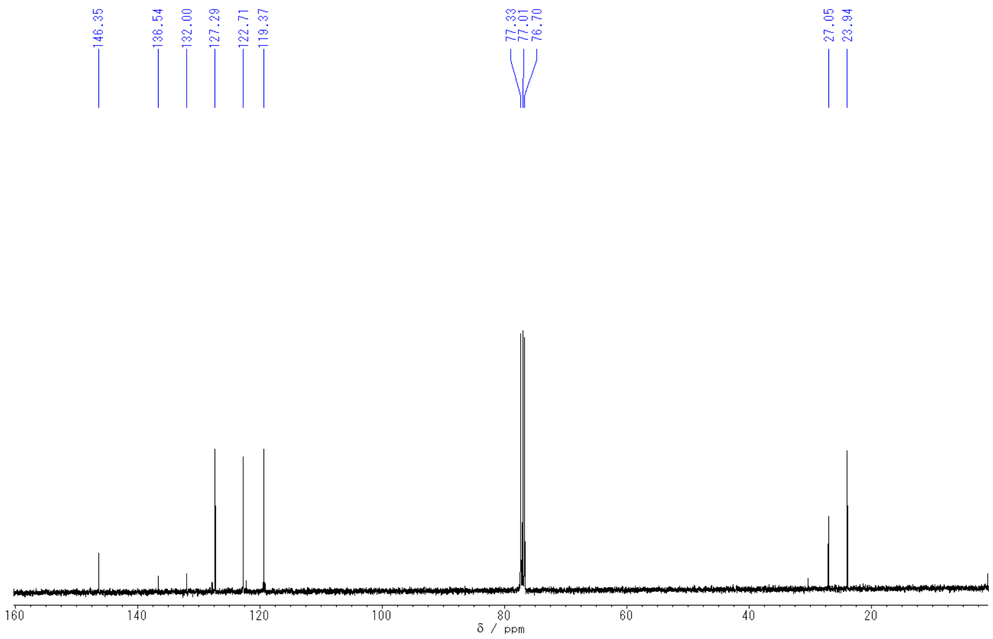

Figure S46. $^{13}C\{^1H\}$ NMR spectrum of **3u** in $CDCl_3$
